# Effects of Intermittent Fasting and Calorie Restriction on Exercise Performance: A Systematic Review and Meta-Analysis

**DOI:** 10.3390/nu17121992

**Published:** 2025-06-13

**Authors:** Fatemeh Kazeminasab, Fatemeh Sharafifard, Ali Bahrami Kerchi, Reza Bagheri, Randhall B. Carteri, Richard Kirwan, Heitor O. Santos, Fred Dutheil

**Affiliations:** 1 Department of Physical Education and Sports Science, Faculty of Humanities, University of Kashan, Kashan 87317-53153, Iran; fatemeh_sharafifard@yahoo.com; 2 Department of Sports Physiology, Faculty of Sports Sciences, Isfahan (Khorasgan) Branch, Islamic Azad University, Isfahan 81551-39998, Iran; alibahramiker11@gmail.com; 3 Department of Exercise Physiology, University of Isfahan, Isfahan 81746-73441, Iran; 4 Methodist University Center, Porto Alegre Institute, Porto Alegre 28311, Brazil; rcarteri@outlook.com; 5 Research Institute for Sports and Exercise Sciences, Liverpool John Moores University, Liverpool L3 3AF, UK; r.p.kirwan@ljmu.ac.uk; 6 Faculdade UNIGUAÇU, Cascavel 85801-180, Brazil; heitoroliveirasantos@gmail.com; 7 Preventive and Occupational Medicine, Université Clermont Auvergne, CNRS, LaPSCo, Physiological and Psychosocial Stress, CHU Clermont-Ferrand, University Hospital of Clermont-Ferrand, Witty Fit, F-63000 Clermont-Ferrand, France; fred_dutheil@yahoo.fr

**Keywords:** intermittent fasting, body composition, calorie restriction, muscle strength, exercise performance

## Abstract

**Context:** Intermittent fasting (IF) and calorie restriction (CR) have gained interest as dietary strategies due to their potential for weight loss and multiple metabolic benefits. These strategies are often accompanied by exercise in an attempt to improve body composition and physical performance. However, further research is crucial to understanding whether or not physical performance is affected by the expected weight loss and related body composition changes in individuals on IF and CR, even when exercise is combined. **Objective:** We aimed to systematically evaluate the effects of IF and CR on exercise performance and body composition in adults aged 18 to 65 years. **Data Source:** A systematic review and meta-analysis of randomized controlled trials (RCTs) was conducted following the Preferred Reporting Items for Systematic Reviews and Meta-Analyses (PRISMA) statement. A systematic review was conducted up to April 2024 by searching electronic databases, including PubMed, Web of Science, and Scopus. There was no limit on publication dates. **Data Extraction:** The search explored the impact of IF and CR combined with exercise vs. exercise alone (control) on exercise performance outcomes: VO_2max_, handgrip strength, bench press strength, knee extensor strength, leg press strength, countermovement jump (CMJ), 400 m walk test, and gait speed; body weight, body mass index (BMI), and body composition: fat-free mass (FFM), fat mass (FM), and body fat percentage (BFP). Analyses included calculation of weighted mean difference (WMD), standardized mean difference (SMD), and 95% confidence intervals (CIs) to assess outcomes. **Data Analysis:** The meta-analysis included a total of 35 studies, ranging from 4 to 52 weeks and involving 1266 participants. The results showed that IF (hypocaloric or eucaloric diet) and CR combined with exercise increased handgrip strength [WMD = 1.707 kg, *p* = 0.01] compared to exercise alone. Moreover, IF and CR combined with exercise did not significantly affect VO_2max_ [SMD = 0.005, *p* = 0.94], bench press strength [WMD = 0.377 kg, *p* = 0.778], knee extensor strength [WMD = −4.729 kg, *p* = 0.12], leg press strength [WMD = −2.874 kg, *p* = 0.415], countermovement jump [WMD = −0.226 cm, *p* = 0.80], 400 m walk test performance [WMD = −8.794 s, *p* = 0.06], or gait speed [WMD = 0.005 m/s, *p* = 0.82] compared to exercise alone. Moreover, IF and CR combined with exercise decreased body weight [WMD = −4.375 kg, *p* = 0.001], BMI [WMD = −1.194 kg·m^−2^, *p* = 0.001], FFM [WMD = −1.653 kg, *p* = 0.001], FM [WMD = −2.858 kg, *p* = 0.001], BFP [WMD = −0.826%, *p* = 0.001] compared to exercise alone. **Conclusions:** IF (hypocaloric or eucaloric) and CR can be effectively integrated into exercise training without negatively impacting most measures of physical performance, while significantly enhancing weight loss and adiposity-related outcomes. The findings from this meta-analysis involving both athletes and non-athletes suggest that weight loss induced by IF and CR combined with exercise does not necessarily result in reduced physical performance. In real-world scenarios, however, different outcomes are conceivable, as body composition, physical capacity, diet and exercise can vary considerably based on individual conditions.

## 1. Introduction

Intermittent fasting (IF) and calorie restriction (CR) have well-documented metabolic benefits [1], but their relevance to sports nutrition and exercise performance remains underexplored. IF, characterized by alternating periods of fasting and eating [2], encompasses protocols such as time-restricted feeding (TRF) [3], alternate-day fasting (ADF) [4], the 5:2 diet [5], and Ramadan fasting [6]. These protocols vary in timing but share the goal of inducing metabolic changes via caloric restriction and meal timing [7]. CR involves sustained calorie reduction without changing meal frequency or compromising nutrition [8]. Both strategies improve insulin sensitivity, lipid profiles, and body composition [9,10]. These strategies are also of interest in sports nutrition, where their effects on performance, strength, endurance, and recovery are being actively studied [11,12].

Both dietary strategies induce physiological changes that may either enhance or impair exercise performance, depending on the circumstances. Fasting conditions promote ketogenesis and alter substrate utilization, favoring fat oxidation over carbohydrate metabolism. This shift may preserve glycogen, benefiting endurance sports [13]. Nonetheless, the reduced energy availability associated with IF and CR may impair performance during high-intensity activities (e.g., sprinting, heavy lifting) or anaerobic exercise, where glycogen is the primary fuel source [2]. Prolonged deficits may impair muscle protein synthesis (MPS) and alter hormones like insulin and insulin-like growth factor-1 (IGF−1), affecting strength, recovery, and training adaptations [14]. In addition to physiological mechanisms, IF and CR may also influence exercise performance through psychological and behavioral pathways. Energy restriction may alter mood, motivation, and perceived exertion, impacting adherence and performance [15]. Rigid timing or caloric limits in some IF/CR protocols may cause disordered eating or reduced exercise enjoyment, especially long-term [16]. These behavioral responses may vary depending on individual factors such as personality traits, previous dieting history, and training experience, further contributing to the heterogeneity in performance outcomes.

The timing of fasting relative to exercise is crucial, as consuming food before or after exercise is essential for optimizing recovery and training outcomes. Training status may influence how IF or CR affects performance. Evidence suggests that well-trained athletes exhibit greater resistance to fasting-induced fatigue, potentially due to superior adaptations in sleep quality, hydration management, and metabolic efficiency, thereby better preserving their performance [17,18,19]. In contrast, amateur or less-trained individuals may experience greater declines in performance, likely driven by increased fatigue from sleep disturbances and dehydration during fasting periods [17]. Nonetheless, further research is warranted to directly compare these populations and to optimize dietary and training strategies accordingly.

Additionally, gender-specific responses to IF and CR have been well-documented in humans. Research shows that women exhibit higher lipid metabolism and lower carbohydrate oxidation during fasting compared to men, a pattern that becomes more pronounced with longer fasting durations (14–38 h) [20]. After a 38 h fast, women show higher plasma free fatty acids and lower glucose, suggesting protection against insulin resistance [21]. The preferential use of lipids by women is largely attributed to estrogen, which promotes lipid oxidation and spares muscle glycogen, thereby altering substrate utilization during energy restriction [20]. These differences highlight the need to consider sex hormones and menstrual cycles, yet many studies overlook them. While these physiological mechanisms may explain the potential effects of IF and CR on exercise performance, their practical implications remain unclear due to inconsistent findings in the scientific literature.

Randomized controlled trials (RCTs) have shown that resistance training combined with TRF compared to a standard diet over 8 weeks, can preserve fat-free mass (FFM) while reducing fat mass (FM), without compromising muscle strength [22,23,24]. However, results from other studies are mixed. While one study found no leg-press strength difference with TRF [23], another reported improved lower-body strength and endurance, and upper-body endurance [22]. A recent study found that one-week fasting reduced VO_2_ peak by 13%, glycogen by ~50%, lean mass by 4.6 kg, and FM by 1.4 kg. However, neither muscle strength (maximal isometric and isokinetic strength) nor oxidative enzymes in skeletal muscle were affected [25]. Notably, one-week fasting did not affect skeletal muscle AMP-activated protein kinase (AMPK) activity, suggesting exercise adaptations may override fasting effects. This study supports the rationale for further investigating and understanding the effects of hypocaloric IF protocols combined with exercise on performance and body composition [25].

Systematic reviews highlight considerable variability in research findings regarding the effects of IF and CR on exercise performance. For instance, a meta-analysis of 28 studies demonstrated that TRF protocols significantly improved maximal oxygen consumption, while Ramadan fasting appeared to impair it. While strength and anaerobic capacity were unaffected, Ramadan fasting reduced aerobic capacity, opposite to TRF’s reported benefits [12]. A review of 25 studies found that IF can enhance body composition, preserve lean mass, and improve maximal power [2]. Nevertheless, a subsequent systematic review and meta-analysis of 11 studies found that Ramadan fasting had a detrimental effect on mean and peak power during high-intensity activities, particularly in the morning, despite having minimal effects on aerobic performance, strength, and jump height [17]. Similarly, CR shows a dichotomy of outcomes. For example, a meta-analysis and meta-regression demonstrated that CR can promote strength gains and increases in lean mass; however, severe caloric deficits greater than 500 kcal/day may compromise muscle strength and recovery [14]. These conflicting findings highlight the need for a comprehensive analysis to determine whether IF and CR are beneficial, neutral, or detrimental to exercise performance.

Most studies on IF and CR are limited by small samples, varied populations, and differing protocols. This heterogeneity has hindered the development of clear, evidence-based guidelines for athletes and practitioners looking to incorporate these dietary strategies into their training regimens. This review aims to consolidate evidence on IF and CR combined with exercise vs. exercise alone, focusing on endurance, strength, and body composition.

## 2. Methods

### 2.1. Trial Registration

The current systematic review and meta-analysis was registered in the PROSPERO International Prospective Register of Systematic Reviews (ID: CRD42024622312; 15 December 2024) and conducted according to The Preferred Reporting Items for Systematic Reviews and Meta-Analyses (PRISMA) guidelines [26], as well as the guidance provided in the Cochrane Handbook of Systematic Reviews of Interventions [27].

### 2.2. Search Strategy

A thorough electronic search was performed across Scopus, Web of Science, and PubMed databases. Two independent reviewers screened published articles up to April 2024, with no restrictions on publication date. The search was confined to English-language articles involving human subjects. Detailed search strategies are provided in Supplementary Table S1. All retrieved records were imported into EndNote, and duplicates were eliminated. Titles, abstracts, and full texts were independently evaluated by two authors for eligibility, with discrepancies resolved through discussion or consultation with a third reviewer. To maximize coverage, reference lists of included studies were also screened for additional relevant articles. Furthermore, supplementary searches were carried out in Google Scholar. The search strategies used for each database are summarized in Supplementary Table S1.

### 2.3. Study Selection and Inclusion Criteria

Studies were included if the intervention duration was ≥4 weeks and involved various types of exercise (aerobic training, high-intensity interval training [HIIT], or resistance training). Eligible studies met the following criteria: (a) peer-reviewed, full-text articles, and (b) trials assessing the effects of IF and CR combined with exercise vs. exercise alone (control) on body weight, VO_2max_, exercise performance (including handgrip strength, bench press strength, knee extensor strength, leg press strength, countermovement jump [CMJ], 400 m walk test, and gait speed), and body composition (including body mass index [BMI], FFM, FM, and body fat percentage [BFP]) in adults aged 18 to 65 years.

**Figure 1:**
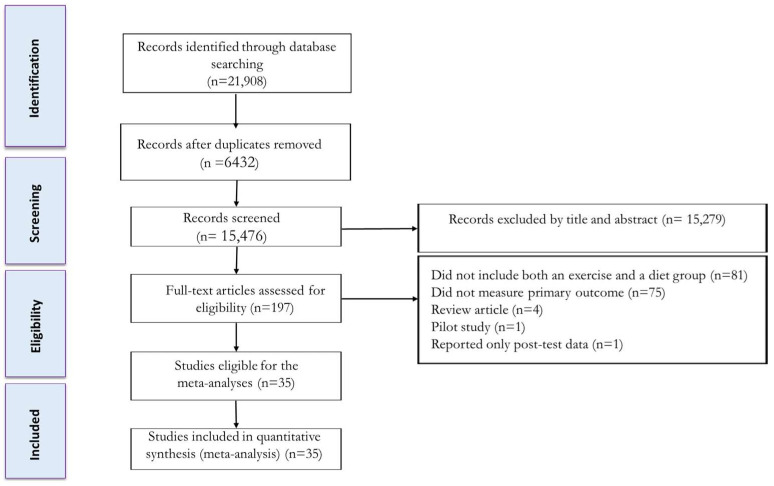
Flow diagram of systematic literature search.

The process for selecting studies is shown in Figure 1. After eliminating duplicate studies, the titles and abstracts of articles were independently evaluated. Then, two reviewers assessed the full texts of potentially eligible studies to determine their eligibility. Any disagreements were resolved through discussion with a third author. The study characteristics extracted included participant details (such as health status, age, biological sex, BMI, and sample size), as well as information on IF, CR, exercise protocols, and intervention duration.

### 2.4. Quality Assessment and Sensitivity Analyses

The assessment of potential bias was conducted using the PEDro (Physiotherapy Evidence Database) tool [28]. Two items were omitted from the original 11-item scale—blinding of participants and blinding of intervention providers—since neither participants nor providers could be blinded to the assigned diet and exercise protocols in the included studies. Consequently, a modified nine-item scale was used in this study, encompassing: (1) clearly defined inclusion and exclusion criteria, (2) randomized allocation of participants, (3) allocation concealment, (4) baseline group comparability, (5) blinding of outcome assessors, (6) outcome assessment in at least 85% of participants, (7) use of intention-to-treat (ITT) analysis, (8) reporting of statistical comparisons between groups, and (9) presentation of point estimates alongside measures of variability (Supplementary Table S2). Sensitivity analyses were performed by systematically excluding one study at a time to evaluate the robustness of the results and the influence of individual studies on the meta-analysis outcomes.

### 2.5. Statistical Analysis

Meta-analyses were conducted using Comprehensive Meta-Analysis version 2.0 (Biostat Inc., Englewood, NJ, USA). Weighted mean difference (WMD), standardized mean difference (SMD), and 95% confidence intervals (CIs) were calculated to evaluate the outcomes. All analyses employed random-effects models. Effect sizes were computed to evaluate and compare the impacts of combining IF and CR with exercise versus exercise alone on body weight, VO_2max_, exercise performance (including handgrip strength, bench press strength, knee extensor strength, leg press strength, CMJ, 400 m walk test, and gait speed), and body composition (BMI, FFM, FM, and BFP). Interpretation of effect sizes was based on the following thresholds: 0.2–0.49 was considered a small effect, 0.5–0.79 a moderate effect, and ≥0.80 a large effect. Heterogeneity among studies was assessed using the I^2^ statistic, with statistical significance set at *p* < 0.05. According to Cochrane guidelines, I^2^ values were interpreted as follows: <25% = low, 25–50% = moderate, 50–75% = high, and ≥75% = considerable heterogeneity [29]. The findings were synthesized using random-effects models to account for potential clinical and methodological heterogeneity that may have influenced the outcomes [30].

Publication bias was assessed through visual inspection of funnel plots. Where asymmetry was detected, Egger’s regression test was conducted as a supplementary confirmatory analysis. A *p*-value of <0.1 was considered indicative of significant publication bias [29].

Subgroup analyses were performed based on age, type of diet (TRF, ADF, Ramadan intermittent fasting [RIF], or CR), intervention duration (short-term intervention ≤8 weeks, or long-term intervention >8 weeks), diet frequency (≤3 days/week, or >3 days/week), type of exercise (aerobic training, resistance training, or combined training), training frequency (low-frequency as ≤3 sessions/week, or high-frequency as >3 sessions/week), intensity of aerobic training (moderate-intensity as 40–80% VO_2max_, or high-intensity as >80% VO_2max_), participant characteristics (trained, or untrained), and gender (female, or male).

## 3. Results

### 3.1. Included Studies

The initial search retrieved a total of 4242 records from PubMed, 11,083 records from Scopus, and 6583 records from Web of Science. After removing duplicate records and assessing the titles and abstracts, 197 studies were deemed relevant and underwent a thorough evaluation of their full texts. Following the full-text assessment, 162 studies were excluded for the following reasons: 81 studies did not include both an exercise and a diet group, 75 studies did not measure primary outcome, 4 studies were review articles, one study was a pilot study, and one study reported only post-test data. As a result, 35 studies were included in this systematic review and meta-analysis, encompassing 39 intervention groups involving the combination of IF and CR with exercise. The flow diagram of the systematic literature search is depicted in Figure 1.

### 3.2. Participant Characteristics

A total of 1266 adults, with or without overweight and obesity, were included, with sample sizes ranging from 15 [31] to 155 [32] participants. The participants’ ages ranged from 18 years [33] to 80 years [34], and BMI values ranged from 21 kg·m^−2^ [35,36] to 35 kg·m^−2^ [37]. A total of 35 studies were included in the analysis. Among them, 13 studies had both male and female participants [32,34,37,38,39,40,41,42,43,44,45,46], 16 studies exclusively included male participants [22,23,31,33,35,36,47,48,49,50,51,52,53,54,55,56], and 6 studies only included female participants [57,58,59,60,61,62]. The participants’ health status varied across the studies: some studies included healthy participants [32,34,37,38,39,40,41,42,43,44,45,46,53,57,58,59,60,61,62], while others focused on participants with overweight and obesity [22,23,31,33,35,36,47,48,49,50,51,52,54,55,56] (Table 1).

### 3.3. Intervention Characteristics

A combination of IF and CR methodologies was implemented across the included studies in the analysis. Six studies used RIF [33,35,48,49,50,51], where participants refrained from eating and drinking from sunrise to sunset. Eleven studies used TRF with a 16 h fasting period an 8 h feeding window [22,23,31,36,43,47,52,54,55,56,60]. Two studies followed a 5:2 diet approach, which involved a 6 h eating window and a fasting period of 18 h on fasting days [40,58]. One study used ADF where participants consumed 25% of their daily recommended energy intake on each fast day [34]. Taken together, IF regimens consisted of either hypocaloric or eucaloric diet. Remaining studies used CR which reduce their daily energy intake by 250–1500 kcal of their habitual diet [32,34,37,38,39,41,42,44,45,46,53,57,59,61,62]. In all cases the control group refrained from IF and consumed a control diet combined with exercise. The length of the interventions ranged from 4 weeks [22,31,33,35,36,46,48,49,50,51,52,55,56,61] to 52 weeks [54,59]. The studies incorporated various forms of exercise training interventions, including high-intensity interval training, aerobic training, resistance training, and power-sports training (specifically, CMJ test). Detailed information on the intervention characteristics can be found in Table 1.

### 3.4. Meta-Analysis

#### 3.4.1. The Effects of IF and CR Combined with Exercise Training on Exercise Performance

##### VO_2max_

**Figure 2:**
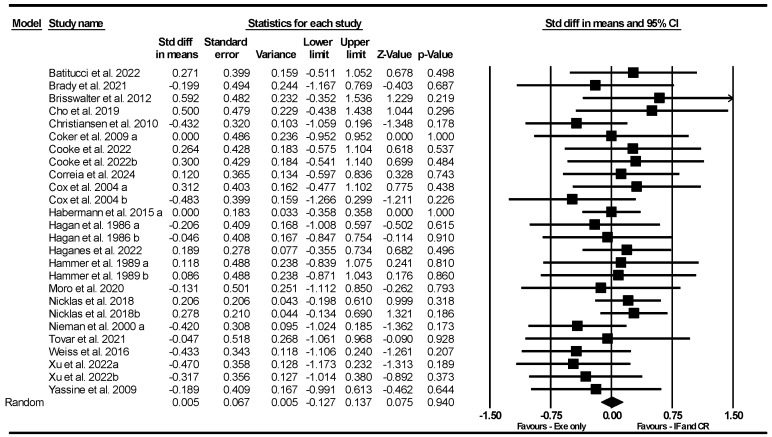
Forest plot of the effects of IF and CR combined with exercise vs. exercise alone on VO_2max_. Data are reported as SMD (95% confidence limits). SMD, standardized mean differences; IF, intermittent fasting; CR, calorie restriction; Exe, exercise [31,32,36,37,38,39,40,41,45,46,47,51,53,56,58,59,60,61,62,63].

Based on 26 intervention arms, the combination of IF and CR with exercise did not significantly alter VO_2max_ [SMD = 0.005 (95% CI −0.127 to 0.137), *p* = 0.94] compared to exercise alone (Figure 2). The studies included in the analysis exhibited no heterogeneity (I^2^ = 0.00%, *p* = 0.84). The absence of publication bias was confirmed by the results of the funnel plots and Egger’s test (*p* = 0.57). Sensitivity analysis, conducted by removing individual studies, showed that the significance and direction of the results remained unchanged.

Subgroup analyses by type of diet showed no significant change in VO_2max_ for TRF [SMD = 0.134 (95% CI −0.145 to 0.412), *p* = 0.34, 8 interventions] and CR [SMD = −0.063 (95% CI −0.216 to 0.091), *p* = 0.42, 16 interventions] compared to exercise alone. One study used RIF and another used ADF.

Subgroup analyses by intervention duration revealed that combining IF or CR with exercise did not significantly affect VO_2max_ compared to exercise alone, either in long-term interventions (>8 weeks) [SMD = −0.023 (95% CI −0.186 to 0.140), *p* = 0.78, 13 interventions] or short-term interventions (≤8 weeks) [SMD = 0.057 (95% CI −0.166 to 0.281), *p* = 0.61, 13 interventions].

Subgroup analyses by diet days/week showed no significant effect on VO_2max_ when combining IF or CR with exercise compared to exercise alone for either >3 days/week [SMD = −0.091 (95% CI −0.253 to 0.071), *p* = 0.27, 19 interventions] or ≤3 days/week [SMD = 0.191 (95% CI −0.035 to 0.417), *p* = 0.09, 7 interventions].

Subgroup analyses by training frequency (number of training sessions per week) revealed that combining IF or CR with exercise did not significantly affect VO_2max_ compared to exercise alone for either >3 sessions/week [SMD = −0.043 (95% CI −0.204 to 0.117), *p* = 0.59, 15 interventions] or ≤3 sessions/week [SMD = 0.104 (95% CI −0.126 to 0.334), *p* = 0.34, 11 interventions].

Subgroup analyses by intensity of aerobic training revealed that combining IF or CR with exercise did not significantly affect VO_2max_ compared to exercise alone for either moderate-intensity aerobic training [SMD = 0.075 (95% CI −0.111 to 0.261), *p* = 0.42, nine interventions] or high-intensity aerobic training [SMD = −0.042 (95% CI −0.384 to 0.299), *p* = 0.80, five interventions].

Subgroup analyses by participant characteristics revealed that combining IF or CR with exercise did not significantly affect VO_2max_ compared to exercise alone in either untrained [SMD = −0.004 (95% CI −0.143 to 0.135), *p* = 0.95, 21 interventions] or trained participants [SMD = 0.082 (95% CI −0.321 to 0.485), *p* = 0.69, 5 interventions].

Subgroup analyses by gender revealed that combining IF or CR with exercise did not significantly affect VO_2max_ compared to exercise alone in either females [SMD = 0.004 (95% CI −0.223 to 0.232), *p* = 0.97, seven interventions] or males [SMD = −0.010 (95% CI −0.312 to 0.293), *p* = 0.95, eight interventions]. Eleven studies included both male and female participants.

##### Handgrip Strength

**Figure 3:**
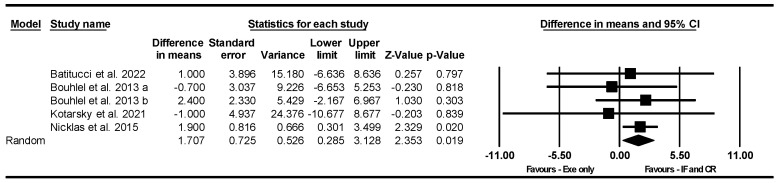
Forest plot of the effects of IF and CR combined with exercise vs. exercise alone on handgrip strength. Data are reported as WMD (95% confidence limits). WMD, weighted mean differences; IF, intermittent fasting; CR, calorie restriction; Exe, exercise [35,43,44,58].

Based on five intervention arms, IF and CR combined with exercise significantly increased handgrip strength [WMD = 1.707 kg (95% CI 0.285 to 3.128), *p* = 0.01] compared to exercise alone (Figure 3). The studies included in the analysis demonstrated no heterogeneity (I^2^ = 0.00%, *p* = 0.89). The absence of publication bias was supported by the results of funnel plots and Egger’s test (*p* = 0.19). Sensitivity analysis, performed by removing individual studies, showed that the significance and direction of the results remained unchanged.

Due to the small number of studies, subgroup analyses based on type of diet, intervention duration, diet days/week, type of exercise, training frequency, and gender were not conducted.

##### Bench Press Strength

**Figure 4:**
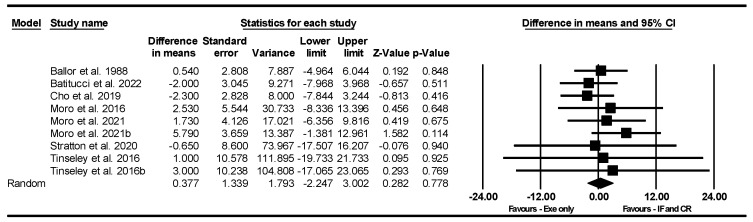
Forest plot of the effects of IF and CR combined with exercise vs. exercise alone on bench press strength. Data are reported as WMD (95% confidence limits). WMD, weighted mean differences; IF, intermittent fasting; CR, calorie restriction; Exe, exercise [22,23,54,55,57,58,63].

Based on 9 intervention arms, IF and CR combined with exercise did not significantly alter bench press strength [WMD = 0.377 kg (95% CI −2.247 to 3.002), *p* = 0.778] compared to exercise alone (Figure 4). The studies included in the analysis showed no heterogeneity (I^2^ = 0.00%, *p* = 0.85). The absence of publication bias was supported by the results of funnel plots and Egger’s test (*p* = 0.44). Sensitivity analysis, conducted by removing individual studies, demonstrated that the significance and direction of the results remained unchanged.

Subgroup analyses by participant characteristics revealed that combining IF or CR with exercise did not significantly affect bench press strength compared to exercise alone in either untrained [WMD = −1.209 kg (95% CI −4.477 to 2.059), *p* = 0.46, three interventions] or trained participants [WMD = 3.259 kg (95% CI −1.146 to 7.664), *p* = 0.14, six interventions].

Due to the limited number of studies, subgroup analyses based on type of diet, intervention duration, diet frequency (days/week), type of exercise, training frequency, and gender were not conducted.

##### Knee Extensor Strength

**Figure 5:**
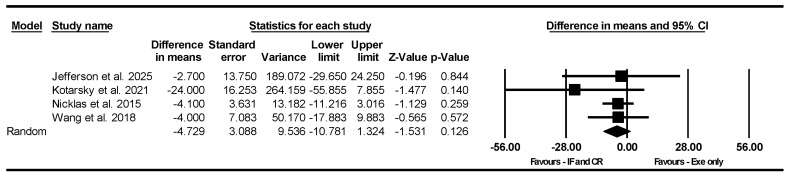
Forest plot of the effects of IF and CR combined with exercise vs. exercise alone on knee extensor strength. Data are reported as WMD (95% confidence limits). WMD, weighted mean differences; IF, intermittent fasting; CR, calorie restriction; Exe, exercise [34,42,43,44].

Based on 4 intervention arms, combining IF and CR with exercise did not significantly alter knee extensor strength [WMD = −4.729 kg (95% CI −10.781 to 1.324), *p* = 0.12] compared to exercise alone (Figure 5). The studies included in the analysis demonstrated no heterogeneity (I^2^ = 0.00%, *p* = 0.69). The absence of publication bias was supported by the results of funnel plots and the Egger’s test (*p* = 0.40). Sensitivity analysis performed by removing individual studies showed that the significance and direction of the results remained unchanged.

Due to the small number of studies, subgroup analyses based on type of diet, intervention duration, diet frequency (days/week), type of exercise, training frequency, and gender were not conducted.

##### Leg Press Strength

**Figure 6:**
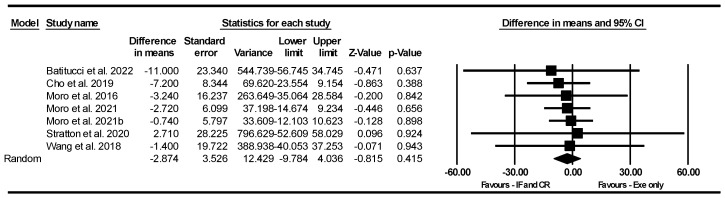
Forest plot of the effects of IF and CR combined with exercise vs. exercise alone on leg press strength. Data are reported as WMD (95% confidence limits). WMD, weighted mean differences; IF, intermittent fasting; CR, calorie restriction; Exe, exercise [23,34,54,55,58,63].

Based on seven intervention arms, combining IF and CR with exercise did not significantly affect leg press strength [WMD = −2.874 kg (95% CI −9.784 to 4.036), *p* = 0.415] compared to exercise alone (Figure 6). The studies included in the analysis demonstrated no heterogeneity (I^2^ = 0.00%, *p* = 0.99). The absence of publication bias was supported by the results of funnel plots and the Egger’s test (*p* = 0.63). Sensitivity analysis, performed by removing individual studies, showed that the significance and direction of the results remained unchanged.

Subgroup analyses by participant characteristics revealed that combining IF or CR with exercise did not significantly affect leg press strength compared to exercise alone in either untrained [WMD = −6.777 kg (95% CI −21.083 to 7.529), *p* = 0.35, three interventions] or trained participants [WMD = −1.686 kg (95% CI −9.578 to 6.205), *p* = 0.67, four interventions].

Due to the small number of studies, subgroup analyses based on type of diet, intervention duration, days of diet per week, type of exercise, training frequency, and gender were not conducted.

##### CMJ

**Figure 7:**
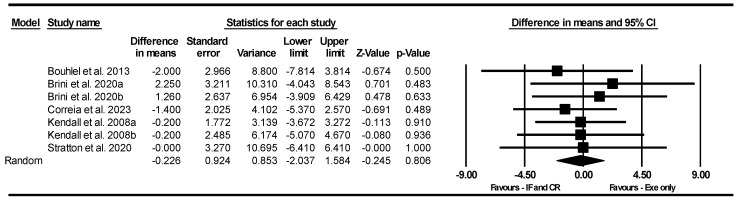
Forest plot of the effects of IF and CR combined with exercise vs. exercise alone on CMJ. Data are reported as WMD (95% confidence limits). WMD, weighted mean differences; IF, intermittent fasting; CR, calorie restriction; Exe, exercise [33,35,50,52,55].

Based on seven intervention arms, IF and CR combined with exercise did not significantly alter CMJ [WMD = −0.226 cm (95% CI −2.037 to 1.584), *p* = 0.80] compared to exercise alone (Figure 7). The studies included in the analysis demonstrated no heterogeneity (I^2^ = 0.00%, *p* = 0.95). The absence of publication bias was confirmed by the results of funnel plots and the Egger’s test (*p* = 0.45). Sensitivity analysis, conducted by removing individual studies, showed that the significance and direction of the results remained unchanged.

Due to the small number of studies, subgroup analyses based on type of diet, intervention duration, days of diet per week, type of exercise, training frequency, and gender were not conducted.

##### The 400 m Walk Test

**Figure 8:**
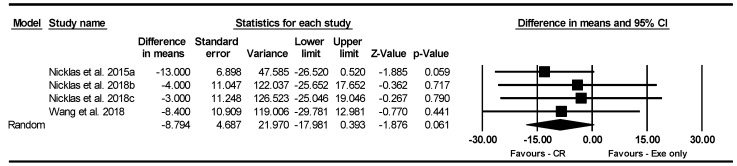
Forest plot of the effects of CR combined with exercise vs. exercise alone on the 400 m walk test. Data are reported as WMD (95% confidence limits). WMD, weighted mean differences; CR, calorie restriction; Exe, exercise [32,34,44].

Based on four intervention arms, IF and CR combined with exercise did not significantly change the 400 m walk test [WMD = −8.794 s (95% CI −17.981 to 0.393), *p* = 0.06] compared to exercise alone (Figure 8). The studies included in the analysis demonstrated no heterogeneity (I^2^ = 0.00%, *p* = 0.84). The absence of publication bias was confirmed by the results of funnel plots and the Egger’s test (*p* = 0.06). Sensitivity analysis, conducted by removing individual studies, showed that the significance and direction of the results remained unchanged.

Due to the small number of studies, subgroup analyses based on type of diet, intervention duration, days of diet per week, type of exercise, training frequency, and gender were not performed.

##### Gait Speed

**Figure 9:**
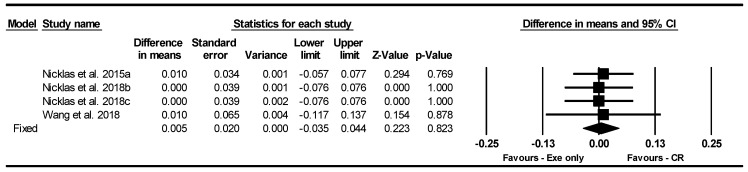
Forest plot of the effects of CR combined with exercise vs. exercise alone on Gait speed. Data are reported as WMD (95% confidence limits). WMD, weighted mean differences; CR, calorie restriction; Exe, exercise [32,34,44].

Based on four intervention arms, CR combined with exercise did not significantly change gait speed [WMD = 0.005 m/s (95% CI −0.035 to 0.044), *p* = 0.82] compared to exercise alone (Figure 9). The studies included in the analysis demonstrated no heterogeneity (I^2^ = 0.00%, *p* = 0.99). The absence of publication bias was supported by the results of funnel plots and the Egger’s test (*p* = 0.88). Sensitivity analysis, performed by removing individual studies, showed that the significance and direction of the results remained unchanged.

Due to the small number of studies, subgroup analyses based on type of diet, intervention duration, days of diet per week, type of exercise, training frequency, and gender were not performed.

#### 3.4.2. The Effects of IF and CR Combined with Exercise Training on Body Weight and Body Composition

##### Body Weight

**Figure 10:**
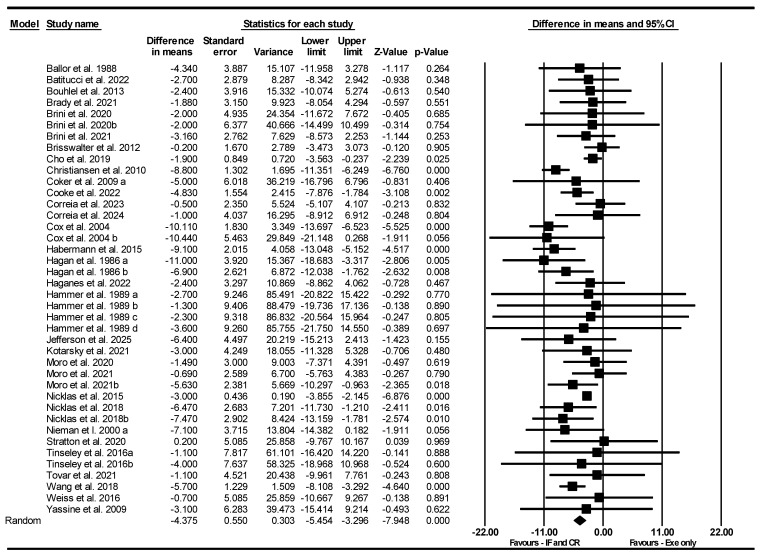
Forest plot of the effects of CR combined with exercise vs. exercise alone on body weight. Data are reported as WMD (95% confidence limits). WMD, weighted mean differences; CR, calorie restriction; Exe, exercise [22,31,32,34,35,36,37,38,39,40,41,42,43,44,45,47,49,50,51,52,53,54,55,56,57,58,59,60,61,62,63].

Based on 40 intervention arms, IF and CR combined with exercise significantly decreased body weight [WMD = −4.375 kg (95% CI −5.454 to −3.296), *p* = 0.001] compared to exercise alone (Figure 10). The studies included in the analysis demonstrated moderate heterogeneity (I^2^ = 41.98%, *p* = 0.003). The absence of publication bias was supported by the results of funnel plots and the Egger’s test (*p* = 0.34). Sensitivity analysis performed by removing individual studies showed that the significance and direction of the results did not change.

Subgroup analyses by type of diet revealed significant reductions in body weight for TRF [WMD = −2.810 kg (95% CI −4.368 to −1.252), *p* = 0.001, 14 interventions] and CR [WMD = −6.425 kg (95% CI −8.459 to −4.792), *p* = 0.001, 20 interventions], but not for RIF [WMD = −1.247 kg (95% CI −3.735 to 1.241), *p* = 0.32, 5 interventions] compared to exercise alone. One study used an ADF diet.

Subgroup analyses by intervention duration revealed that combining IF or CR with exercise led to significant reductions in body weight compared to exercise alone, both in long-term interventions > 8 weeks [WMD = −6.738 kg (95% CI −8.583 to −4.893), *p* = 0.001, 18 interventions] and short-term interventions ≤8 weeks [WMD = −2.053 kg (95% CI −3.100 to −1.006), *p* = 0.001, 22 interventions].

Subgroup analyses by days of diet/week revealed that combining IF or CR with exercise led to significant reductions in body weight compared to exercise alone, both for >3 days/week [WMD = −4.432 kg (95% CI −5.709 to −3.156), *p* = 0.001, 35 interventions] and ≤3 days/week [WMD = −3.974 kg (95% CI −6.185 to −1.764), *p* = 0.001, 5 interventions].

Subgroup analyses by type of exercise revealed that combining IF or CR with exercise led to significant reductions in body weight compared to exercise alone, both for aerobic training [WMD = −5.117 kg (95% CI −6.587 to −3.646), *p* = 0.001, 28 interventions], resistance training [WMD = −3.238 kg (95% CI −4.001 to −2.476), *p* = 0.001, 10 interventions], and combined training [WMD = −1.942 kg (95% CI −3.573 to −0.311), *p* = 0.001, 2 interventions].

Subgroup analyses by training frequency revealed that combining IF or CR with exercise led to significant reductions in body weight compared to exercise alone, both for high-frequency training > 3 sessions/week [WMD = −5.515 kg (95% CI −7.213 to −3.817), *p* = 0.001, 18 interventions] and low-frequency training ≤ 3 sessions/week [WMD = −3.899 kg (95% CI −5.292 to −2.506), *p* = 0.001, 20 interventions].

Subgroup analyses by participant characteristics revealed that combining IF or CR with exercise led to significant reductions in body weight compared to exercise alone, both in trained [WMD = −1.649 kg (95% CI −3.238 to −0.060), *p* = 0.04, 15 interventions] and untrained participants [WMD = −5.609 kg (95% CI −7.027 to −4.192), *p* = 0.001, 25 interventions].

Subgroup analyses by gender revealed that combining IF or CR with exercise led to significant reductions in body weight compared to exercise alone, both in female [WMD = −5.966 kg (95% CI −8.172 to −3.760), *p* = 0.001, 10 interventions] and male participants [WMD = −3.333 kg (95% CI −5.325 to −1.340), *p* = 0.001, 18 interventions]. Thirteen studies included both male and female participants.

##### BMI

**Figure 11:**
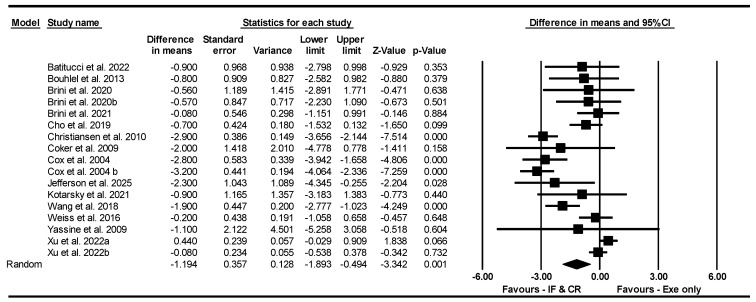
Forest plot of the effects of CR combined with exercise vs. exercise alone on BMI. Data are reported as WMD (95% confidence limits). WMD, weighted mean differences; CR, calorie restriction; Exe, exercise [34,35,37,38,39,42,43,45,46,49,50,53,58,63].

Based on 17 intervention arms, IF and CR combined with exercise decreased BMI [WMD = −1.194 kg·m^−2^ (95% CI −1.893 to −0.494), *p* = 0.001] significantly compared to exercise alone (Figure 11). The studies included in the analysis demonstrated high heterogeneity (I^2^ = 86.66%, *p* = 0.001). The absence of publication bias was supported by the results of funnel plots and the Egger’s test (*p* = 0.16). Sensitivity analysis performed by removing individual studies showed that the significance and direction of the results did not change.

Subgroup analyses by type of diet revealed significant reductions in BMI for CR [WMD = −1.569 kg·m^−2^ (95% CI −2.591 to −0.547), *p* = 0.003, 10 interventions], but not for TRF [WMD = −0.900 kg·m^−2^ (95% CI −2.359 to 0.559), *p* = 0.22, 2 interventions], nor RIF [WMD = −0.364 kg·m^−2^ (95% CI −1.124 to 0.395), *p* = 0.34, 4 interventions] compared to exercise alone. One study used an ADF diet.

Subgroup analyses by intervention duration revealed that combining IF or CR with exercise led to significant reductions in BMI compared to exercise alone, both in long-term interventions > 8 weeks [WMD = −1.569 kg·m^−2^ (95% CI −2.591 to −0.547), *p* = 0.003, 10 interventions] and short-term interventions ≤ 8 weeks [WMD = −0.566 kg·m^−2^ (95% CI −1.090 to −0.043), *p* = 0.03, 7 interventions].

Subgroup analyses by days of diet/week revealed that combining IF or CR with exercise led to significant reductions in BMI compared to exercise alone, for >3 days/week [WMD = −1.571 kg·m^−2^ (95% CI −2.333 to −0.810), *p* = 0.001, 13 interventions], but not for ≤3 days/week [WMD = −0.090 kg·m^−2^ (95% CI −0.623 to 0.443), *p* = 0.74, 4 interventions].

Subgroup analyses by type of exercise revealed that combining IF or CR with exercise led to significant reductions in BMI compared to exercise alone, both for aerobic training [WMD = −1.123 kg·m^−2^ (95% CI −1.982 to −0.264), *p* = 0.01, 13 interventions] and resistance training [WMD = −1.962 kg·m^−2^ (95% CI −2.768 to −1.156), *p* = 0.001, 2 interventions], but not for combined training [WMD = −0.723 kg·m^−2^ (95% CI −1.505 to 0.058), *p* = 0.07, 2 interventions].

Subgroup analyses by training frequency revealed that combining IF or CR with exercise led to significant reductions in BMI compared to exercise alone, for low-frequency ≤3 sessions/week [WMD = −1.603 kg·m^−2^ (95% CI −2.449 to −0.757), *p* = 0.001, eight interventions], but not for high-frequency > 3 sessions/week [WMD = 0.062 kg·m^−2^ (95% CI −0.232 to 0.356), *p* = 0.67, seven interventions].

Subgroup analyses by participant characteristics revealed that combining IF or CR with exercise led to significant reductions in BMI compared to exercise alone in untrained [WMD = −1.400 kg·m^−2^ (95% CI −2.239 to −0.561), *p* = 0.001, 13 interventions], but not in trained participants [WMD = −0.364 kg·m^−2^ (95% CI −1.124 to 0.395), *p* = 0.34, 4 interventions].

Due to the small number of studies, subgroup analyses based on gender were not performed.

##### FFM

**Figure 12:**
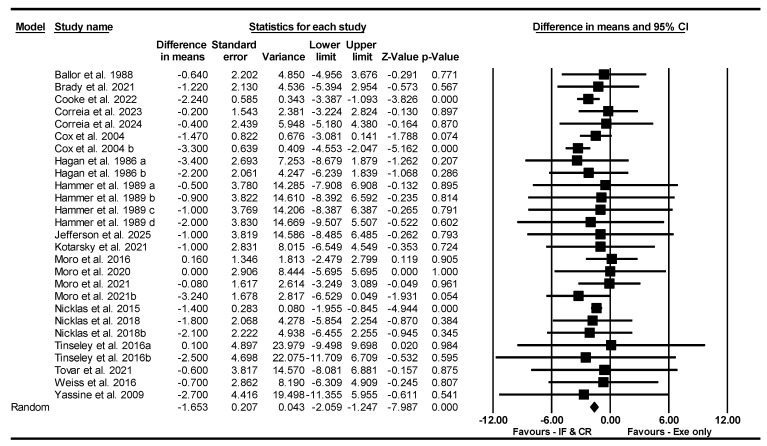
Forest plot of the effects of CR combined with exercise vs. exercise alone on fat-free mass. Data are reported as WMD (95% confidence limits). WMD, weighted mean differences; CR, calorie restriction; Exe, exercise [22,23,31,32,36,37,40,41,42,43,44,45,47,52,53,54,56,57,61].

Based on 27 intervention arms, IF and CR combined with exercise decreased FFM [WMD = −1.653 kg (95% CI −2.059 to −1.247), *p* = 0.001] significantly compared to exercise alone (Figure 12). The studies included in the analysis demonstrated no heterogeneity (I^2^ = 0.00%, *p* = 0.95). The absence of publication bias was supported by the results of funnel plots and the Egger’s test (*p* = 0.51). Sensitivity analysis performed by removing individual studies showed that the significance and direction of the results did not change.

Subgroup analyses by type of diet revealed significant reductions in FFM for TRF [WMD = −1.537 kg (95% CI −2.380 to −0.695), *p* = 0.001, 12 interventions] and CR [WMD = −1.688 kg (95% CI −2.151 to −1.225), *p* = 0.001, 15 interventions] compared to exercise alone. One study used an ADF diet.

Subgroup analyses by intervention duration revealed that combining IF or CR with exercise led to significant reductions in FFM compared to exercise alone, both for long-term interventions > 8 weeks [WMD = −1.736 kg (95% CI −2.199 to −1.274), *p* = 0.001, 13 interventions] and short-term interventions ≤ 8 weeks [WMD = −1.376 kg (95% CI −2.219 to −0.533), *p* = 0.001, 14 interventions].

Subgroup analyses by days of diet/week revealed that combining IF or CR with exercise led to significant reductions in FFM compared to exercise alone, both for >3 days/week [WMD = −1.561 kg (95% CI −2.00 to −1.123), *p* = 0.001, 24 interventions] and ≤3 days/week [WMD = −2.201 kg (95% CI −3.271 to −1.131), *p* = 0.001, 3 interventions].

Subgroup analyses by type of exercise revealed that combining IF or CR with exercise led to significant reductions in FFM compared to exercise alone, both for aerobic training [WMD = −2.249 kg (95% CI −2.915 to −1.584), *p* = 0.001, 17 interventions] and resistance training [WMD = −1.303 kg (95% CI −1.817 to −0.789), *p* = 0.001, 9 interventions]. One study had a combined training protocol.

Subgroup analyses by training frequency revealed that combining IF or CR with exercise led to significant reductions in FFM compared to exercise alone, for low-frequency ≤ 3 sessions/week [WMD = −1.492 kg (95% CI −1.945 to −1.040), *p* = 0.001, 13 interventions], but not for high-frequency > 3 sessions/week [WMD = −1.181 kg (95% CI −2.518 to 0.155), *p* = 0.08, 13 interventions].

Subgroup analyses by participant characteristics revealed that combining IF or CR with exercise led to significant reductions in FFM compared to exercise alone, for untrained [WMD = −1.761 kg (95% CI −2.189 to −1.333), *p* = 0.001, 17 interventions], but not for trained participants [WMD = −0.701 kg (95% CI −1.974 to 0.571), *p* = 0.28, 10 interventions].

Subgroup analyses by gender revealed that combining IF or CR with exercise led to significant reductions in FFM compared to exercise alone, for males [WMD = −1.916 kg (95% CI −2.693 to −1.139), *p* = 0.001, 13 interventions], but not for females [WMD = −1.327 kg (95% CI −3.638 to 0.985), *p* = 0.26, 6 interventions]. Eight studies had both male and female participants.

##### FM

**Figure 13:**
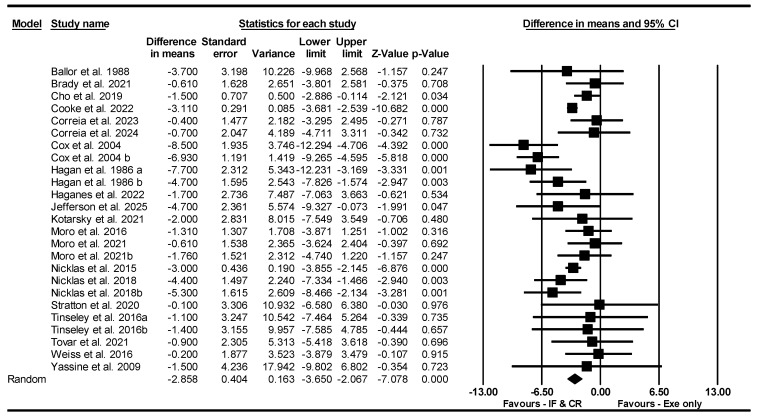
Forest plot of the effects of CR combined with exercise vs. exercise alone on fat mass. Data are reported as WMD (95% confidence limits). WMD, weighted mean differences; CR, calorie restriction; Exe, exercise [22,23,31,32,37,40,41,42,43,44,45,47,52,53,54,55,56,57,60,63].

Based on 25 intervention arms, IF and CR combined with exercise decreased FM [WMD = −2.858 kg (95% CI −3.650 to −2.067), *p* = 0.001] compared to exercise alone (Figure 13). The studies included in the analysis demonstrated no heterogeneity (I^2^ = 50.44%, *p* = 0.002). The absence of publication bias was supported by the results of funnel plots and the Egger’s test (*p* = 0.72). Sensitivity analysis performed by removing individual studies showed that the significance and direction of the results did not change.

Subgroup analyses by type of diet revealed significant reductions in FM for TRF [WMD = −2.540 kg (95% CI −3.109 to −1.971), *p* = 0.001, 13 interventions] and CR [WMD = −4.716 kg (95% CI −6.203 to −3.229), *p* = 0.001, 11 interventions] compared to exercise alone. One study used an ADF diet.

Subgroup analyses by intervention duration revealed that combining IF or CR with exercise led to significant reductions in FM compared to exercise alone, for long-term interventions > 8 weeks [WMD = −4.460 kg (95% CI −5.912 to −3.009), *p* = 0.001, 11 interventions] and short-term interventions ≤ 8 weeks [WMD = −2.095 kg (95% CI −2.784 to −1.406), *p* = 0.001, 14 interventions].

Subgroup analyses by days of diet/week revealed that combining IF or CR with exercise led to significant reductions in FM compared to exercise alone, for >3 days/week [WMD = −2.708 kg (95% CI −3.803 to −1.614), *p* = 0.001, 21 interventions] and ≤3 days/week [WMD = −3.069 kg (95% CI −4.372 to −1.765), *p* = 0.001, 4 interventions].

Subgroup analyses by type of exercise revealed that combining IF or CR with exercise led to significant reductions in FM compared to exercise alone, for aerobic training [WMD = −3.791 kg (95% CI −5.161 to −2.420), *p* = 0.001, 13 interventions], resistance training [WMD = −2.487 kg (95% CI −3.192 to −1.781), *p* = 0.001, 10 interventions] and combined training [WMD = −1.529 kg (95% CI −2.874 to −0.185), *p* = 0.02, 2 interventions].

Subgroup analyses by training frequency revealed that combining IF or CR with exercise led to significant reductions in FM compared to exercise alone, for high-frequency > 3 sessions/week [WMD = −3.101 kg (95% CI −4.739 to −1.463), *p* = 0.001, 9 interventions] and low-frequency ≤ 3 sessions/week [WMD = −2.446 kg (95% CI −3.249 to −1.644), *p* = 0.001, 15 interventions].

Subgroup analyses by participant characteristics revealed that combining IF or CR with exercise led to significant reductions in FM compared to exercise alone, for untrained [WMD = −3.804 kg (95% CI −4.778 to −2.830), *p* = 0.001, 15 interventions], but not for trained participants [WMD = −0.933 kg (95% CI −2.067 to 0.200), *p* = 0.10, 10 interventions].

Subgroup analyses by gender revealed that combining IF or CR with exercise led to significant reductions in FM compared to exercise alone, for females [WMD = −3.902 kg (95% CI −6.382 to −1.422), *p* = 0.002, 3 interventions] and males [WMD = −2.583 kg (95% CI −4.349 to −0.817), *p* = 0.004, 13 interventions]. Nine studies had both male and female participants.

##### BFP

**Figure 14:**
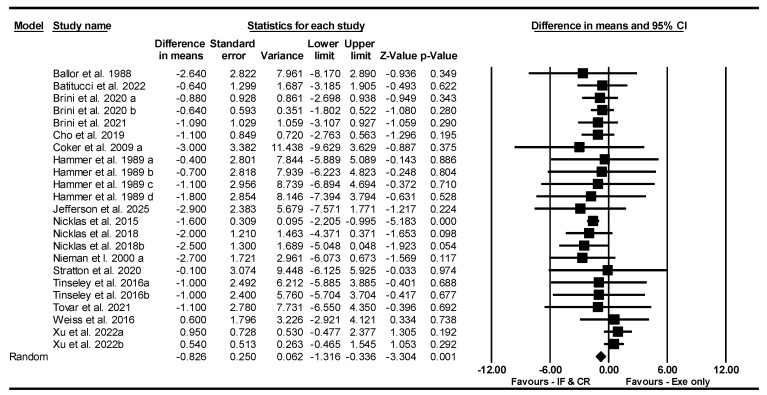
Forest plot of the effects of CR combined with exercise vs. exercise alone on body fat percentage. Data are reported as WMD (95% confidence limits). WMD, weighted mean differences; CR, calorie restriction; Exe, exercise [22,32,39,42,44,45,46,49,50,55,56,57,58,61,62,63].

Based on 23 intervention arms, IF and CR combined with exercise decreased BFP [WMD = −0.826% (95% CI −1.316 to −0.336), *p* = 0.001] compared to exercise alone (Figure 14). The studies included in the analysis demonstrated no heterogeneity (I^2^ = 14.14%, *p* = 0.26). The absence of publication bias was supported by the results of funnel plots and the Egger’s test (*p* = 0.82). Sensitivity analysis performed by removing individual studies showed that the significance and direction of the results did not change.

Subgroup analyses by type of diet revealed no significant changes in BFP for TRF [WMD = −0.746% (95% CI −2.563 to 1.072), *p* = 0.42, 5 interventions], CR [WMD = −0.930% (95% CI −1.864 to 0.001), *p* = 0.05, 14 interventions], and RIF [WMD = −0.782 (95% CI −1.663 to 0.099), *p* = 0.08, 3 interventions] compared to exercise alone. One study used an ADF diet.

Subgroup analyses by intervention duration revealed that combining IF or CR with exercise led to significant reductions in BFP compared to exercise alone for short-term interventions ≤ 8 weeks [WMD = −0.866% (95% CI −1.564 to −0.167), *p* = 0.01, 12 interventions], but not for long-term interventions >8 weeks [WMD = −0.921% (95% CI −1.962 to 0.120), *p* = 0.08, 11 interventions].

Subgroup analyses by days of diet/week revealed that combining IF or CR with exercise led to significant reductions in BFP compared to exercise alone for >3 days/week [WMD = −0.429% (95% CI −0.617 to −0.242), *p* = 0.001, 17 interventions], but not for ≤3 days/week [WMD = −0.157% (95% CI −0.497 to 0.182), *p* = 0.36, 6 interventions].

Subgroup analyses by type of exercise revealed that combining IF or CR with exercise led to significant reductions in BFP compared to exercise alone for aerobic training [WMD = −0.222% (95% CI −0.404 to −0.039), *p* = 0.01, 16 interventions] and resistance training [WMD = −0.480% (95% CI −0.854 to −0.105), *p* = 0.01, 6 interventions].

Subgroup analyses by training frequency revealed that combining IF or CR with exercise led to significant reductions in BFP compared to exercise alone for high-frequency > 3 sessions/week [WMD = −0.202% (95% CI −0.403 to −0.001), *p* = 0.04, 12 interventions] and low-frequency ≤ 3 sessions/week [WMD = −0.513% (95% CI −0.753 to −0.273), *p* = 0.001, 10 interventions].

Subgroup analyses by participant characteristics revealed that combining IF or CR with exercise led to significant reductions in BFP compared to exercise alone for untrained [WMD = −0.305% (95% CI −0.521 to −0.089), *p* = 0.006, 16 interventions], but not for trained participants [WMD = −0.276% (95% CI −0.619 to 0.067), *p* = 0.11, 7 interventions].

Subgroup analyses by gender revealed that combining IF or CR with exercise led to no significant changes in BFP compared to exercise alone for females [WMD = −0.290% (95% CI −0.606 to 0.027), *p* = 0.07, seven interventions], or males [WMD = −0.276% (95% CI −0.619 to 0.067), *p* = 0.11, seven interventions]. Nine studies included both male and female participants.

### 3.5. Quality Assessment

The PEDro tool was used to assess the methodological quality of each individual study, with scores ranging from 4 to 7 out of a possible maximum of 9 points. Two studies had a score of 4 [53,57], 12 studies had scores of 5 [22,33,35,37,38,47,51,55,56,58,59,61], 14 studies had scores of 6 [31,34,39,41,42,43,45,48,49,50,52,60,62], 7 studies had scores of 7 [23,32,36,40,44,46,54]. Most of the PEDro scores were lowered due to two items (concealed allocation, and intention-to-treat analysis). The details of the quality analysis are shown in Supplementary Table S2.

## 4. Discussion

This meta-analysis explored the effects of combining IF (both hypocaloric and eucaloric) and CR with exercise on endurance performance, strength, and body composition. The findings suggest that integrating IF and CR into exercise programs offers notable benefits, particularly in weight loss and improvements in adiposity, without negatively impacting exercise performance. Specifically, while IF and CR paired with exercise did not significantly enhance VO_2max_, aerobic performance, gait speed, or upper- and lower-body strength, a minor increase in handgrip strength was observed; however, the clinical relevance of this change (−1.7 kg) remains unclear, as it is unlikely to contribute to significant functional improvements in this context. However, the analysis indicated that combining IF and CR with exercise resulted in greater reductions in body weight, BFP, and FM compared to exercise alone, while also reducing FFM (−1.65 kg, *p* = 0.001), which is an unfavorable outcome.

FFM, which includes muscle mass in addition to water, bones, minerals, proteins, organs, and other lean tissues [64], is often lost along with FM during a caloric deficit, particularly in situations where dietary protein intake is insufficient to preserve lean tissue. Typically, approximately 20–40% of total weight loss can be attributed to FFM, with the remaining portion coming from FM [65]. Correspondingly, we observed nearly twice the reduction in FM (−2.86 kg) compared to FFM. Given the difficulty in accessing muscle mass directly, a substantial portion of the FFM loss was likely attributable to water. It is well-established that during the initial days of a caloric deficit, total body water loss can account for 1–2 kg, which may represent up to ~70% of the early weight loss. This is largely due to the depletion of glycogen stores, approximately 500 g of glycogen (~400 g in muscle and ~100 g in the liver) with each gram of glycogen binding roughly 4 g of water. Over time, as the deficit becomes more chronic, the body shifts toward losing primarily FM and protein, with estimates suggesting that ~85% of the total body weight loss derives from fat and ~15% from protein [65,66]. Considering glycogen as a component of FFM, avoiding CR and carbohydrate depletion in the lead-up to competition may be advisable for athletes [67]. Preserving or even increasing glycogen stores through carbohydrate supercompensation is a well-supported strategy to optimize performance, irrespective of the specific carbohydrate source or timing [68,69,70]. In this context, our meta-analysis highlights the potential for strategically implementing hypocaloric IF combined with exercise as part of a broader nutrition periodization plan.

The preservation of FFM during IF and exercise remains debated, as IF can induce a caloric deficit that, if not managed with adequate protein intake, may impair FFM maintenance. The balance between energy intake and expenditure is crucial for preserving muscle mass while achieving fat loss. Both exercise and feeding promote mechanistic target of rapamycin (mTOR) pathway signaling, which activates several biosynthetic pathways. However, factors such as protein intake, the magnitude of the caloric deficit, and daily energy expenditure, which are inconsistently reported in studies, also likely influence FFM preservation [14]. Notably, trained individuals experience less significant body composition changes compared to untrained individuals, as their body composition is more strongly influenced by training-related energy expenditure rather than by diet alone [71]. This suggests that the impact of dietary interventions may be less pronounced in individuals with a well-established exercise regimen. More specifically, a daily protein intake of 1.7–2.2 g/kg has been suggested to mitigate FFM loss due to its structural role in skeletal muscle, particularly in those undergoing energy restriction or exercise [72]. Recent studies suggest that myofibrillar protein synthesis can be preserved under IF and CR, which is a validated measure of acute muscle hypertrophy. This preservation of protein synthesis may play a role in maintaining FFM during energy restriction, as increased protein turnover helps prevent the loss of skeletal muscle [73,74]. A typical IF regimen (e.g., TRF with an eight-hour eating window) does not reduce daily rates of myofibrillar protein synthesis, a validated measure of acute muscle hypertrophy, when compared to an isocaloric diet with equivalent protein content [73]. Finally, short-term (10 days) ADF and CR did not impair MPS rates, despite similar losses of FFM and FM, when both diets were compared to an energy-balanced control diet with matched protein intake, at least in middle-aged males with overweight or obesity [74]. This finding suggests that, under certain conditions, IF and CR may not have detrimental effects on MPS rates.

Although we observed greater improvements in weight loss and adiposity with IF and CR combined with exercise compared to exercise alone, the overall impact of IF regimens on body weight and FM loss is similar to other diets with matched energy intake [75,76,77]. However, IF combined with exercise may offer additional benefits related to metabolic flexibility and adherence to dietary regimens. The decrease in energy intake remains the primary mechanism for inducing weight loss; however, appetite management is crucial, with an estimated increase in energy intake of 100 kcal/day per kilogram of weight lost [78]. No wonder that long-term success in preventing weight regain remains a challenge in both modern pharmacotherapy and non-pharmacological strategies involving diet and exercise [79,80,81]. There is hope that IF may result in less hunger compared to regular meals; however, on the flip side, IF can also sharply trigger hunger. A systematic review of 16 studies showed that TRE (daily eating window of ≤12 h) did not lead to significant increases in hunger or other appetite-related measures, but it was associated with higher appetite at bedtime [82]. Skipping breakfast can be considered a form of TRE; however, it presents a dilemma when it comes to weight loss [83,84]. A high-calorie breakfast and low-calorie dinner increased the thermic effect of food by a factor of 2.5 compared to a calorie-equated diet with a high-calorie dinner and low-calorie breakfast in normal-weight men, suggesting that meal timing may influence energy expenditure and metabolic processes. This highlights the potential metabolic benefits associated with chrononutrition [85]. However, introducing breakfast earlier in women who regularly skipped breakfast resulted in an increase in energy intake (~270 kcal), as well as a rise in body weight by 600 g and FM by 500 g over four weeks compared to a group that continued to skip breakfast [86].

Exercise intervention alone is not a strong inducer of FM loss, as the extent of fat loss often depends on the combination of exercise with dietary changes. While exercise increases energy expenditure, it may not be sufficient to achieve substantial FM reductions without dietary adjustments. For example, common resistance training interventions result in an estimated loss of 600 g of FM [87], while typical aerobic training protocols, including those of moderate and high intensity, are associated with a loss of 1 to 3 kg of FM [88]. Epidemiologically, total energy expenditure plateaus in the higher ranges of physical activity, supporting a constrained total energy expenditure model and highlighting the challenge of weight loss even in physically active individuals [89], after adjusting for body size and composition. Although resistance training is more specific for muscle hypertrophy and strength [90,91,92], some level of aerobic training can also partially enhance muscle hypertrophy and related metabolic pathways [93,94]. Although our meta-analysis encompassed various exercise protocols, combined or not, and recognizing that all types of exercise can provide benefits to body composition and performance, the most effective non-pharmacological strategy for addressing obesity outcomes is to combine CR (either through regular meals or IF regimens) with a mix of aerobic and resistance training. If only one option must be chosen, resistance training is preferred. In addition, maintaining a proper protein intake (at least 1.1–1.7 g/kg/body weight) and a moderate caloric deficit of 500–1000 kcal are key nutritional targets for optimizing body recomposition [95].

When evaluating performance, the lack of significant changes in key parameters from combining IF or CR with exercise aligns with recent literature. For instance, Conde-Pipó et al. concluded that IF does not negatively impact sports performance and could be a viable dietary strategy for athletes, with potential performance declines being more likely attributed to factors such as inadequate rest and hydration [2]. Conversely, a recent systematic review and meta-analysis examining TRF over 4–8 weeks reported positive effects on weight loss and cardiometabolic variables, although negative effects were observed during Ramadan fasting [12]. These conclusions were based on 11 studies involving 485 participants; however, high heterogeneity among the studies warrants caution in interpretation.

It is important to note that IF and CR combined with exercise increased handgrip strength by approximately 1.7 kg compared to exercise alone, a result that is clinically questionable and lacks specificity depending on the type of sport and its relevance in clinical settings. The absence of adequate blinding in dietary interventions, similar to what is commonly used for medications or supplements, as well as the small number of studies (five), may influence the outcome. Epidemiologically, a 1 kg increase in handgrip strength is associated with a 3.2% reduction in all-cause mortality and a 6% reduction in respiratory mortality among older individuals [96,97]. However, while some studies in our meta-analysis included older adults, most focused on younger individuals without serious clinical concerns.

Although handgrip strength has been shown to be a predictor of upper, lower, and total strength, a recent systematic review reveals variability in the correlations between handgrip strength and muscle torque. Therefore, caution should be exercised when using handgrip strength alone as a measure. The neutral effects we observed for other strength and functional parameters outweigh the clinically questionable increase in handgrip strength. Mechanistically, changes in body composition, cardiovascular function, and metabolic parameters may contribute to performance improvements [98]. However, many studies have failed to replicate the metabolic changes observed in animal models, suggesting that species-specific differences may limit the direct translation of animal model findings to human populations [99]. More specifically, the positive effects on handgrip strength may be attributed to other variables. For instance, baseline differences in body weight and BMI between the exercise and IF groups, as observed in the study by Batitucci et al. [58] could explain the variations in handgrip strength adaptations [100]. Additionally, reductions in adiposity may positively influence strength outcomes [101], as suggested by Nicklas et al. [44] and Bouhlel et al. [35], though further research is required to confirm these findings. Overall, the evidence suggests that IF and CR regimens do not significantly impact exercise performance, making them suitable for integration into personalized and periodized nutrition plans without compromising performance [102].

The consistent finding of enhanced weight and fat loss when combining IF and CR with exercise is supported by recent literature. For instance, Correa et al. reported that TRF regimens were associated with significant weight loss (WMD: −1.07 kg, 95% CI: −1.74 to −0.40; *p* = 0.002) and reduced fasting glucose levels (WMD: −1.71 mg/dL, 95% CI: −3.20 to −0.21; *p* = 0.03), particularly in RCTs. Similarly, our analysis of 27 interventions found significant FFM loss when combining IF and CR with exercise, irrespective of exercise type. However, Keenan et al. [103] concluded that FFM is preserved when IF is combined with resistance training, although their review only included studies where exercise was performed in the fed state and lacked a control diet group. The mechanisms underlying the weight-lowering effects of IF and CR with exercise are multifaceted. For example, fasted-state exercise enhances fat oxidation compared to the fed state, driven by differences in glycemia and insulinemia [104]. Additionally, IF combined with exercise increases AMPK signaling, activating fat-oxidation pathways involving proteins such as fatty acid translocase/cluster of differentiation 36 (FAT-CD36), carnitine palmitoyltransferase 1 (CPT1), and pyruvate dehydrogenase kinase 4 (PDK4) [105,106,107], and citrate synthase, which improve intramuscular triacylglycerol and fatty acid utilization [99,108]. While the long-term regulation of these mechanisms remains understudied, they may explain the positive effects observed in both short- and long-term interventions. Exercise intensity also plays a role, with low- to moderate-intensity fasted exercise (45–65% VO_2max_) enhancing lipid metabolism, whereas high-intensity exercise (85% VO_2max_) shows no significant differences [109].

This study has several strengths, including a comprehensive evaluation of IF regimens combined with exercise across multiple outcomes and a rigorous selection of studies through a systematic review. This approach helps reduce bias and heterogeneity, ensuring that the findings are robust and reliable. In addition to the various metabolic benefits of IF and CR in both athletic and clinical settings [102,110,111,112,113,114], our findings expand knowledge by providing practical insights for both athletes and non-athletes regarding exercise performance and body composition. Specifically, we demonstrate that combining IF and CR diets with exercise did not impair performance while effectively reducing body weight and adiposity, suggesting positive physiological adaptations. However, variations in participant health characteristics, such as baseline body composition, and differences in intervention durations may have influenced the results. While this analysis evaluated numerous health and performance outcomes, the limited number of studies available for subgroup analyses based on exercise type, duration, and intensity underscores the need for further research. Lastly, while IF is generally considered safe, caution should be exercised in individuals with diabetes, particularly those on insulin therapy, as disturbances in blood glucose may occur if not properly monitored. This is especially relevant during exercise, as changes in glucose utilization could affect performance and overall safety [115]. Such a concern is particularly relevant when IF is combined with CR and exercise, as lower carbohydrate intake and increased carbohydrate oxidation are expected.

## 5. Conclusions

This meta-analysis underscores the practical implications of IF and caloric CR for both athletes and non-athletes. Our findings demonstrate that IF (whether hypocaloric or eucaloric) and CR can be effectively integrated with exercise training without compromising performance. Furthermore, when the primary goal is to improve body composition, combining IF or CR with exercise appears to be a viable strategy for reducing body weight and adiposity, although strategies should be implemented to limit losses in FFM. These results highlight the flexibility of IF and CR as dietary approaches that can be tailored to individual needs and training goals. However, further research is needed to explore the long-term effects, optimal protocols, and individual variability in response to these interventions.

## Figures and Tables

**Table 1:** Study, participant, and intervention characteristics.

Source, Year	Study Characteristics				Participant Characteristics			Intermittent Fasting/Calorie Restriction Characteristics	Non-Fasting/Non-Calorie Restriction Control Eating	Exercise Characteristics	Energy Intake (kcal/Day)	Hypocaloric Diet/Eucaloric Diet
	Sample Size (Sex)	Groups	Intervention Duration (Weeks)	Outcomes	Health Status	Age (Years) Mean ± SD	BMI (kg·m^−2^) Mean ± SD					
Ballor et al., 1988 [57]	20 F	Exe + CR Exe alone	8	Chest press strength FM FFM BFP BW	Obesity	Exe + CR 32.9 ± 1.5 Exe alone: 32.9 ± 1.5	Exe + CR: - Exe alone: -	Energy needs were estimated based on body metrics and activity level, then reduced by 1000 kcal/day. The final intake averaged 2200–2500 kcal/day. The diet provided 50% carbohydrate, 27% protein, 23% fat, and included a daily protein supplement.	In the Exe alone group, participants were asked to keep their usual dietary intake unchanged.	Participants performed bench press, leg press, lat pulldown, biceps curl, triceps extension, calf raise, leg extension, and hamstring curl, following the protocol by Sprague and Reynolds. Each exercise was performed three times per week: 2 sets of 10 reps, followed by a third set to voluntary fatigue	A daily caloric deficit of 1000 kcal was applied to baseline needs, estimated at 2200–2500 kcal/day.	Hypocaloric diet
Batitucci et al., 2022 [58]	26 F	TRF + Exe Exe alone	8	Leg press strength Chest press strength Handgrip strength VO_2max_ BFP BMI BW	Obesity	TRF + Exe: 32.2 ± 4.4 Exe alone: 33 ± 3.0	TRF + Exe: 34 ± 3.2 Exe alone: 31.8 ± 2.02	The 5:2 intermittent fasting protocol was implemented twice weekly on non-consecutive days over 8 weeks. Each fasting day followed a 6:18 schedule, allowing two meals within a 6 h window, followed by 18 h of complete fasting	The Exe alone group was instructed to maintain their habitual diet during the intervention.	HIIT was performed three times per week for 8 weeks, with each 225 min session including a 4 min warm-up, 18 min of intervals at 70–85% HRmax (30–45 s effort, 15–30 s recovery), and a 3 min cool-down.	1700–1800 kcal/day	Hypocaloric diet
Bouhlel et al., 2013 [35]	20 M	RIF + Exe Exe alone	4	Vertical jump Handgrip strength BMI BW	Healthy participants (physical education students) Trained	RIF + Exe: 21.8 ± 1.9 Exe alone: 21.8 ± 1.9	RIF + Exe: 22.4 ± 1.9 Exe alone: 21.4 ± 2.2	During Ramadan, participants consumed their last meal between 8:00 and 9:00 p.m. and fasted daily from 5:00 a.m. to approximately 6:30 p.m.	NR	They engaged in 16 h per week of training across various sports, including soccer, handball, basketball, volleyball, gymnastics, and athletics.	NR	Hypocaloric diet (10% reduction in calorie intake)
Brady et al., 2021 [47]	17 M	TRF + Exe Exe alone	8	VO_2_peak FM FFM BW	Healthy participants (Middle and long-distance runners) Trained	TRF + Exe: 35.9 ± 8.6 Exe alone: 39.9 ± 3	TRF + Exe: NR Exe alone: NR	Participants were directed to eat all meals within an 8 h period, usually from 12:00 to 20:00, with only water allowed outside this window.	Participants were advised to maintain their usual dietary habits throughout the intervention period.	Participants were required to be current competitors in middle- and long-distance events (≥1500 m) and to self-report training a minimum of five days weekly.	The average daily energy intake in the TRF group declined by 5.2 ± 3.6 kcal/kg/day from PRE to MID.	Hypocaloric diet
Brini et al., 2019 [48]	16 M	RIF + SSG SSG RIF + RSA RSA	4	SJ CMJ BFP BMI	Healthy participants (Basketball players) Trained	RIF + SSG: 23.4 ± 2.3 SSG: 23.4 ± 2.3 RIF + RSA: 23.4 ± 2.3 RSA: 23.4 ± 2.3	RIF + SSG: 22.6 ± 1.95 SSG: 22.6 ± 1.95 RIF + RSA: 22.6 ± 1.95 RSA: 22.6 ± 1.95	During the Ramadan phase, participants fasted from dawn until sunset, abstaining from both food and drink.	Participants maintained stable nutrition and hydration routines, consuming customary Iftar and Sahur meals in proximity to each testing session.	Players from the same basketball team followed SSG and RSA protocols based on their group allocation (GSSG and GRSA), with sessions scheduled at least 24 h apart, twice per week.	NR	Eucaloric diet
Brini et al., 2020 [49]	24 M	RIF + Exe Exe alone	4	BFP BMI BW	Healthy participants (Basketball players) Trained	RIF + Exe: 25.32 ± 2.56 Exe alone: 24.85 ± 1.55	RIF + Exe: 23.43 ± 1.19 Exe alone: 23.41 ± 1.46	Participants fasted for roughly 16–17 h each day.	Participants documented all meals consumed during the study, including the quantity and type of food and beverages. Additionally, a nutritionist conducted interviews and analyzed their records.	Training sessions began with a 115 min warm-up, followed by technical and tactical drills focusing on fundamental and basketball-specific movements, including defense. Both groups completed approximately 90 min per session, three times per week.	RIF + Exe: 2741.66 ± 172.98 kcal/day Exe alone: 2829.17 ± 73.04 kcal/day	Hypocaloric diet (20% reduction in calorie intake)
Brini et al., 2021 [50]	24 M	RIF + Exe Exe alone	4	BMI BW	Professional male basketball players	RIF + Exe: 25.32 ± 2.56 Exe alone: 24.85 ± 1.55	RIF + Exe: 23.43 ± 1.19 Exe alone: 23.41 ± 1.46	Ramadan intermittent fasting	Normal diet	Training sessions started with a 15 min warm-up program, followed by technical and tactical drills based on basic basketball movements, specific basketball movements, and basic defensive movements, Both groups completed the same training volume (~90 min per session). 3 day/week	RIF + Exe: 2741.66 ± 172.98 Exe alone: 2829.17 ± 73.04	Eucaloric diet
Brisswalter et al., 2011 [51]	18 M	RIF + Exe Exe alone	4	VO_2max_ BW	Healthy participants (well-trained runners) Trained	RIF + Exe: 23.6 ± 2.9 Exe alone: 23.6 ± 2.9	RIF + Exe: NR Exe alone: NR	Ramadan intermittent fasting	Normal diet	Training consisted of (1) 30 min of slow running followed by intervals of 30 s running and 30 s rest at maximal aerobic speed (100% MAS), (2) a continuous 30 min run at 100% MAS, and (3) 20 min of slow running at the athlete’s individual competition speed, conducted three times weekly.	NR	Eucaloric diet
Cho et al., 2019 [63]	18 M&F	ADF + Exe Exe alone	8	Chest press strength Leg press strength VO_2max_ FM BFP BMI BW	Obesity	ADF + Exe: 34.5 ± 5.7 Exe alone: 38.6 ± 8.2	ADF + Exe: 28 ± 2.6 Exe alone: 26.9 ± 3.9	During the 8-week intervention, ADF + Exe participants consumed about 25% of their daily energy needs (~500 kcal) on each fast day (24 h) and ate freely on feed days (24 h). Fast and feed days alternated every other day, with fast days occurring three times weekly. On fast days, participants were instructed to eat a single meal between 12 p.m. and 2 p.m. to standardize fasting periods	Control group participants were instructed to maintain their usual diet.	Resistance training sessions lasted 40 min and involved weight machines, barbells, and dumbbells. Intensity was tailored to each participant’s muscle strength and progressively increased weekly. Aerobic training consisted of 20 min on motorized treadmills, with intensity set according to individual maximal oxygen consumption. Both training types were performed three times per week.	ADF + Exe: 1785.9 ± 311.3 kcal/day Exe alone: 1532.8 ± 410.4 kcal/day	Hypocaloric diet
Christiansen et al., 2010 [38]	40 M&F	CR + Exe Exe alone	12	VO_2max_ BMI BW	Obesity	CR + Exe: 37.5 ± 8 Exe alone: 37.2 ± 7	CR + Exe: 34.2 ± 3 Exe alone: 33.4 ± 4	Participants in the CR + Exe group were assigned a daily intake of 800 kcal.	NR	Participants completed supervised aerobic training three times weekly, each session lasting 60–75 min, with an estimated energy expenditure of 500–600 kcal per session.	Participants in the DIO and DEX groups 600 and 800 kcal/day for 8 weeks followed by a weight maintenance diet for 4 weeks. The participants in the DEX group were allowed to consume 150–200 kcal more per day compared with the DIO group	Hypocaloric diet
Coker et al., 2009 [39]	17 M&F	CR + Exe Exe alone	12	VO_2_peak BFP BMI BW	Obesity	CR + Exe: 54 ± 6 Exe alone: 55 ± 5.64	CR + Exe: 32 ± 3 Exe alone: 32 ± 2.82	Caloric restriction began with a 1000 kcal reduction in the first week, followed by weekly decreases of 500 kcal until reaching a total weekly deficit of 2500 kcal.	NR	Training intensity was prescribed at 50% of participants’ peak oxygen consumption.	NR	Hypocaloric diet
Cooke et al., 2022 [40]	22 M&F	TRF + Exe Exe alone	8 16	VO_2_peak FM FFM BW	Obesity	TRF + Exe: 39 ± 6.8 Exe alone: 32 ± 8.3	TRF + Exe: 34 ± 2.1 Exe alone: 32 ± 4.4	The 5:2 IF group restricted energy intake on two non-consecutive days per week for 16 weeks, consuming ad libitum on the other five days. On fasting days, men and women consumed a single meal of 600 kcal and 500 kcal, respectively, along with unlimited water and unsweetened black tea.	NR	SIT involved supervised sessions of 4 × 20 s efforts at 150% VO_2_peak, each followed by 40 s of active rest at 50 watts on a magnetic braked cycle ergometer. The number of intervals increased from four to six during the first four weeks and stayed at six thereafter. Sessions included 3 min warm-up and cool-down periods, performed three times per week.	NR	Hypocaloric diet
Correia et al., 2023 [52]	18 M	TRF + Exe Exe alone	4	CMJ SJ FM FFM BW	Healthy participants Trained	TRF + Exe: 23.7 ± 2.6 Exe alone: 23.7 ± 2.6	TRF + Exe: NR Exe alone: NR	During TRF, participants ate two to three ad libitum meals within an 8 h window (1–9 p.m.), consuming only water, tea, and black coffee outside this period.	During the Exe-only condition, participants consumed 100% of their energy requirements and maintained their usual dietary habits.	Participants followed a structured training program during each dietary intervention, performing four sets of 8–10 repetitions at 85% of their 1-RM on leg press, bench press, leg extension, leg curl, shoulder press, and lat pulldown, three times per week.	Before TRF + Exe: 2433.3 ± 760.5 kcal/day Before Exe alone: 2427 ± 556.8 kcal/day	Eucaloric diet
Correia et al., 2024 [31]	15 M	TRF + Exe Exe alone	4	VO_2max_ FM FFM BW	Healthy participants Trained	TRF + Exe: 23.7 ± 2.6 Exe alone: 23.7 ± 2.6	TRF + Exe: Exe alone: NR	The TRF intervention used a 16/8 protocol, where participants consumed 2 to 3 meals within an 8 h window (1:00–9:00 p.m.) and were allowed only water, tea, and black coffee outside this period.	During the Exe-only condition, participants were asked to maintain their usual dietary habits without any restrictions on meal timing throughout the intervention.	During the first 2 weeks of the intervention, participants completed 10 km runs per session with 24 h of rest between workouts. In the final 2 weeks, they continued the 10-km runs and added 1 km running intervals after each continuous run, separated by 4 min of active recovery, totaling approximately 39 km per week. Sessions were performed three times per week.	TRF + Exe: 2311 ± 681 kcal/day Exe alone: 2285 ± 659 kcal/day	Eucaloric diet
Cox et al., 2004 [53]	30 M	CR + High intensity Exe High intensity Exe CR + Low intensity Exe Low intensity Exe	16	VO_2max_ FM FFM BMI BW	Overweight participants	CR + High intensity Exe: 41.1 ± 5.4 High intensity Exe: 43 ± 4.2 CR + Low intensity Exe: 41.1 ± 5.4 Low intensity Exe: 43 ± 4.2	CR + High intensity Exe: 33.3 ± 3.6 High intensity Exe: 30.5 ± 3.9 CR + Low intensity Exe: 33.3 ± 3.6 Low intensity Exe: 30.5 ± 3.9	Participants decreased their daily energy intake by 1000 to 1500 kcal.	Control participants were instructed to keep their regular dietary habits.	The exercise consisted solely of stationary cycling, with participants cycling for 30 min at 60–70% of their maximum workload, as determined by their fitness assessment. Each session included a 5 min warm-up and a 5-min cool-down, performed three times per week.	Participants in the other two groups followed personalized programs targeting a daily energy intake reduction of 1000 to 1500 kcal.	Hypocaloric diet
Habermann et al., 2015 [59]	120 F	CR + Exe Exe alone	52	VO_2max_ BW	Postmenopausal women with obesity/overweight	CR + Exe: 58.4 ± 4.7 Exe alone: 59.1 ± 5.1	CR + Exe: 31.2 ± 4.5 Exe alone: 30.5 ± 3.6	The diet targeted a daily energy intake of 1200–2000 kcal, adjusted by weight, with less than 30% of calories from fat. The goal was to achieve a 10% weight loss within 6 months, followed by maintenance.	Participants were instructed to maintain their usual dietary habits.	The exercise intervention aimed for 45 min of moderate-to-vigorous activity. Participants attended three supervised sessions per week and exercised twice weekly at home. Training duration increased from 15 min at 60–70% of maximal heart rate to 70–85% by week 7, which was then maintained. Facility sessions included treadmill walking, stationary cycling, and other aerobic machines, performed five days per week.	The diet had a total energy intake goal of 1200–2000 kcal/day based on weight	Hypocaloric diet
Hagan et al., 1986 [41]	24 M 24 F	CR + Exe (male) Exe alone (male) CR + Exe (female) Exe alone (female)	12	VO_2max_ FM FFM BW	Overweight	M CR + Exe: 34.4 ± 5.6 Exe alone: 33.9 ± 7.6 F CR + Exe: 34.2 ± 6.5 Exe alone: 37.2 ± 7.4	NR	Intake was limited to 1200 kcal per day.	Participants continued their usual dietary habits.	Aerobic training was performed for 30 min per session, five days per week.	NR	Hypocaloric diet
Haganes et al., 2022 [60]	52 F	TRF + Exe Exe alone	7	VO_2_peak FM BW	Obesity	TRF + Exe: 37.3 ± 5.7 Exe alone: 34.9 ± 7	TRF + Exe: 31.4 ± 4 Exe alone: 32.5 ± 4.5	In the TRF + Exe group, participants restricted their energy intake to a maximum 10 h eating window each day.	NR	Participants engaged in three supervised treadmill workouts per week.	During the baseline week, total daily energy intake averaged 2066 ± 484 kcal in the TRF group, 1996 ± 387 kcal in the Exe alone group, 1943 ± 346 kcal in the TRF + Exe group, and 2056 ± 433 kcal in the Exe alone group.	Eucaloric diet
Hammer et al., 1989 [61]	20 F	CR + Exe Exe alone	4 16	VO_2_max FFM BFP BW	Obesity	CR + Exe: 33 ± 8 Exe alone: 32 ± 7	CR + Exe: 32.2 ± 7.8 Exe alone: 31.3 ± 6.7	Participants in the CR + Exe group followed an 800 kcal/day exchange list diet, with specified servings for each food group. The CR diet was set at an 800 kcal daily intake.	No effort was made to restrict calories in the Exe group	Participants started the first week by walking or jogging 1.6 km per session. The distance increased by 0.8 km each week until reaching 4.8 km, which was maintained for the rest of the study. Exercise intensity was set between 70 and 85% of HRmax, performed five days per week.	The estimated caloric deficits were 1203 kcal/day	Hypocaloric diet
Jefferson et al., 2016 [42]	32 M&F	CR + Exe Exe alone	20	Knee extensor strength FM FFM BFP BMI BW	Obesity	CR + Exe: 69 ± 3 Exe alone: 68 ± 3	CR + Exe: 30.7 ± 2.5 Exe alone: 31 ± 2.7	Participants were guided to decrease their daily energy intake by about 600 kcal below estimated expenditure, aiming for a 5–10% weight loss.	The Exe alone group was asked to maintain their usual diet during the intervention.	Upper and lower body exercises were performed on different machines, with intensity set at 70% of 1RM. Exercise volume and intensity gradually increased over the first month, conducted three times per week.	NR	Hypocaloric diet
Kirkendall et al., 2008 [33]	85 M	RIF + Exe Exe alone	4	Vertical jump	Healthy participants (young football players) Trained	RIF + Exe: 18 Exe alone: 18	RIF + Exe: 22.4 Exe alone: 22.4	Participants were instructed to adhere to fasting protocols throughout the month of Ramadan.	Participants were instructed not to fast during that period.	NR	NR	Eucaloric diet
Kotarsky et al., 2021 [43]	21 M&F	TRF + Exe Exe alone	8	Handgrip strength Knee extensor strength FM FFM BMI BW	Obesity	TRF + Exe: 45 ± 3 Exe alone: 44 ± 2	TRF + Exe: 29.8 ± 0.8 Exe alone: 29.4 ± 0.8	TRF + Exe participants consumed all calories daily between 12:00 p.m. and 8:00 p.m., creating a 16 h fasting window. During fasting, TRF participants were advised to drink only water, black coffee, or tea.	Exe alone participants were instructed to keep their usual eating patterns.	Resistance training included three sets of 12 reps with up to 60 s rest between sets. Load was based on a percentage of body weight and adjusted via trial loads, performed three times per week. Aerobic training consisted of 75 min at moderate-to-vigorous intensity (≥55% HRR) on a treadmill or similar equipment. Intensity was increased by 5–10% during weeks 5 and 7 to ensure progression. Sessions were performed immediately after resistance training, three times per week.	A modest but significant energy restriction occurred from pre-intervention to week 7, with approximately 300 kcal/day (14.5%) in the TRF group and 250 kcal/day (11.4%) in the Exe group.	Hypocaloric diet
Moro et al., 2016 [23]	34 M	TRF + Exe Exe alone	8	Chest press strength Leg press strength FM FFM	Healthy participants (Resistance trained) Trained	TRF + Exe: 29.94 ± 4.07 Exe alone: 28.47 ± 3.48	TRF + Exe: NR Exe alone: NR	TRF participants met 100% of their energy needs through three meals at 1 p.m., 4 p.m., and 8 p.m., fasting for the remaining 16 h each day.	The Exe alone group distributed their daily caloric intake across three meals, which were consumed at approximately 8:00 a.m., 1:00 p.m., and 8:00 p.m.	Participants engaged in a split resistance training routine, completing three weekly sessions. Each session comprised three sets of 6–8 reps at 85–90% of 1-RM, performed to failure with 3 min rest intervals between sets and exercises.	TRF: 2826 ± 412.3 kcal/day, carbohydrates 53.2 ± 1.4%, fat 24.7 ± 3.1%, protein 22.1 ± 2.6%, CON: 3007 ± 444.7 kcal/day, carbohydrates 54.7 ± 2.2%, fat 23.9 ± 3.5%, protein 21.4 ± 1.8%	Eucaloric diet
Moro et al., 2020 [36]	16 M	TRF + Exe Exe alone	4	VO_2_peak FFM BW	Healthy participants (Elite cyclists) Trained	TRF + Exe: 19.38 ± 2.39 Exe alone: 19.38 ± 1.60	TRF + Exe: 21.85 ± 1.65 Exe alone: 22.47 ± 1.83	Participants in the TRF group were advised to consume their entire daily caloric requirement across four eating occasions, including the three main meals, within an 8 h feeding window.	Participants in the Exe group consumed their daily caloric intake following a conventional meal timing schedule.	This investigation was carried out during the winter pre-competition phase. The athletes followed a structured training regimen comprising approximately 500 ± 50 km per week, distributed across six sessions, all conducted within the designated feeding period (10:00 a.m. to 6:00 p.m.).	All participants adhered to an identical 7-day dietary regimen, with a fixed daily caloric intake of 4800 kcal.	Eucaloric diet
Moro et al., 2021 [54]	20 M	TRF + Exe Exe alone	8 52	Chest press strength Leg press strength FM FFM BW	Healthy participants Trained	TRF + Exe: NR Exe alone: NR	TRF + Exe: NR Exe alone: NR	The TRF group was instructed to meet their total daily energy requirements through three meals consumed within an 8 h eating window (approximately at 1 p.m., 4 p.m., and 8 p.m.), followed by a 116 h fasting period.	Participants in the Exe group followed their usual meal pattern, consuming their total daily energy intake across three meals spread over an approximately 12 h period (~8 a.m., 1 p.m., and 8 p.m.).	Throughout the experimental period, training intensity varied between 75% and 90% of 1 RM to cycle between strength and hypertrophy phases. Sessions were scheduled between 4:00 and 6:00 p.m. to align with the eating window for both groups, occurring three times per week.	In the TRF group, energy intake was allocated as 40% at breakfast, 25% at lunch, and 35% at dinner, while participants following a normal diet (ND) consumed 25%, 40%, and 35%, respectively. Additionally, all participants ingested 20 g of whey protein 30 min post-exercise.	Eucaloric diet
Nicklas et al., 2015 [44]	111 M&F	CR + Exe Exe alone	20	Handgrip strength Knee extensor strength 400 m walk test Gait speed FM FFM BFP BW	Overweight and obesity	CR + Exe: 69.6 ± 3.9 Exe alone: 69.4 ± 3.6	CR + Exe: 30.4 ± 2.2 Exe alone: 30.7 ± 2.4	Each participant received an individualized daily caloric target, calculated by deducting 600 kcal from their estimated energy requirements for weight maintenance. Up to two meal replacements per day (shakes and bars; Slim-Fast Inc.)—each providing approximately 220 kcal with 7–10 g protein, 33–46 g carbohydrates, 1.5–5 g fat, and 2–5 g fiber—were supplied for breakfast and lunch.	Participants in the resistance training only group were instructed to maintain a eucaloric diet throughout the intervention.	The training protocol aimed for participants to perform 3 sets of 10 repetitions per exercise at 70% of their one-repetition maximum (1 RM). Rest intervals between sets were approximately one minute. Resistance was progressively increased when a participant successfully completed 10 repetitions in the third set across two consecutive sessions. Strength assessments were conducted every four weeks to recalibrate training loads, ensuring alignment with the 70% 1 RM target. Training sessions occurred three times per week.	NR	Hypocaloric diet
Nicklas et al., 2019 [32]	155 M&F	Mod-CR + Exe High-CR + Exe Exe alone	20	VO_2_peak 400 m walk test Gait speed FM FFM BFP BW	Obesity	Mod-CR + Exe: 68.8 ± 3.1 High-CR + Exe: 69.6 ± 3.8 Exe alone: 69.1 ± 3.7	Mod-CR + Exe: 34.7 ± 3.7 High-CR + Exe: 34.4 ± 3.7 Exe alone: 34.6 ± 3.1	Participants in the calorie restriction (CR) groups received two meals per day (lunch and dinner), collected thrice weekly. Their prescribed caloric intake was calculated by reducing their estimated daily energy requirements for weight maintenance by either 250 kcal (moderate CR) or 600 kcal (high CR).	Participants in the Exe alone group were instructed to continue their usual dietary habits throughout the intervention.	Aerobic training was conducted on treadmills, beginning with a slow-paced walking warm-up. Training duration increased from 15 to 20 min at 50% heart rate reserve (HRR) during the first week to 30 min at 65–70% HRR by week six. Sessions were performed four times per week.	No female participant received less than 1100 kcal/day, and no male participant received less than 1300 kcal/day.	Hypocaloric diet
Nieman et al., 2000 [62]	43 F	CR + Exe Exe alone	12 weeks	VO_2_max BFP BW	Obesity	CR + Exe: 45.6 ± 1.1 Exe alone: 43.2 ± 2.3	CR + Exe: 33.1 ± 0.62 Exe alone: 33.1 ± 0.62	Participants with obesity followed a 4.19 to 5.44 MJ/day (1200 to 1300 kcal) diet for 12 weeks. The meal plan was structured using dietary exchanges, including two servings of fruit, three of vegetables, two of dairy, six of grains, two of fats, five of lean protein, and 100 kcal of optional foods.	NR	Participants were instructed to walk for 45 min per session at 60% to 80% of their maximum heart rate over a 12-week period, totaling 60 exercise sessions. Exercise duration and intensity were progressively increased during the initial 3 weeks, starting from 25 to 30 min per session at 60% to 65% MHR in week one, reaching 45 min at 70% to 80% MHR from weeks 4 to 12. Sessions were conducted five days per week.	1884 ± 84 kcal/day	Hypocaloric diet
Stratton et al., 2020 [55]	26 M	TRF + Exe Exe alone	4	Vertical Jump Chest press strength Leg press strength FM BFP BW	Healthy participants Trained	TRF + Exe: 22.9 ± 3.6 Exe alone: 22.5 ± 2.2	TRF + Exe: NR Exe alone: NR	The TRF + Exe group consumed all calories and macronutrients within an 8 h daily window while adhering to a prescribed 25% caloric deficit.	Exe alone group followed their usual daily feeding schedule while maintaining a prescribed 25% caloric deficit.	Resistance training sessions included 1–2 min of rest between sets. Exercise loads were determined based on pre-intervention estimated 1 RM percentages, and all exercises were performed within a specified repetitions-in-reserve range. Training occurred three times per week.	NR	Hypocaloric diet
Tinsley et al., 2017 [22]	18 M	TRF + Exe Exe alone	4 8	Chest press strength FM FFM BFP BW	Healthy participants Trained	TRF + Exe: 22.9 ± 4.1 Exe alone: 22.0 ± 2.4	TRF + Exe: NR Exe alone: NR	On non-training days (four days per week), participants were instructed to consume all their calories within a flexible four-hour window between 4:00 p.m. and midnight, without restrictions on calorie amount or food types.	Participants in the Exe alone group were instructed to maintain their habitual dietary habits throughout the study.	Participants performed alternating upper and lower body workouts, completing four sets of 8–12 repetitions per exercise, with 90 s of rest between sets, three times per week.	TRF + Exe: 1631 ± 563 kcal/day Exe alone: 2318 ± 977 kcal/day	Eucaloric diet
Tovar et al., 2021 [56]	15 M	TRF + Exe Exe alone	4	VO_2max_ HR FM FFM BFP BW	Healthy participants Trained	TRF + Exe: 28.7 ± 5.2 Exe alone: 28.7 ± 5.2	TRF + Exe: NR Exe alone: NR	The 16/8 protocol required participants to consume all meals within a consistent 8 h daily window. During the fasting period, only water and non-caloric beverages such as unsweetened black coffee or plain tea were permitted.	Under the 12/12 protocol, participants were instructed to consume all their calories within a consistent, self-chosen 12 h daily window.	The weekly schedule consisted of one day with high-intensity exercise, one day with moderate-intensity exercise, one day with low-intensity exercise, and one rest day, totaling three exercise sessions per week.	TRF + Exe: 2421 ± 478 kcal/day Exe alone: 2513 ± 367 kcal/day	Eucaloric diet
Wang et al., 2019 [34]	26 M&F	CR + Exe Exe alone	20	400 m walk test Gait Speed Leg press strength Knee extensor strength BMI BW	Obesity	CR + Exe: 65–80 Exe alone: 65–80	CR + Exe: 29.7 ± 1.8 Exe alone: 29.7 ± 2.2	Participants assigned to the CR + Exe group followed a dietary weight-loss protocol aimed at achieving moderate weight reduction of 5–10%.	Participants were instructed to maintain their habitual dietary intake.	One-repetition maximum (1 RM) was used to determine training intensity on weight-stack resistance machines. The protocol aimed for participants to perform 3 sets of 10 repetitions at 70% of their 1 RM, with approximately 1 min of rest between sets. Resistance was increased once a participant successfully completed 10 repetitions on the third set in two consecutive sessions. Training occurred three times per week.	NR	Hypocaloric
Weiss et al., 2016 [45]	35 M&F	CR + Exe Exe alone	12	VO_2max_ FM FFM BFP BMI BW	Overweight participants	CR + Exe: 57 ± 7 Exe alone: 56 ± 6	CR + Exe: 28.3 ± 1.8 Exe alone: 27 ± 1.5	All three interventions aimed to induce a 20% energy deficit, targeting a body weight reduction of 6–8% over 12 to 14 weeks (approximately 0.5% of baseline body weight lost per week). The duration of the intervention was extended for individual participants as necessary to achieve the prescribed weight loss goal.	Participants were instructed to maintain their usual dietary intake.	The objective of the Exe intervention was to increase total energy expenditure (TEE) by approximately 20% without altering dietary habits. Recommended activities included cardiovascular exercises such as brisk walking and cycling, as well as functional physical activities like walking to work or carrying out yard work, performed six days per week.	CR + Exe: 2310 ± 159 Exe alone: 1908 ± 125	Hypocaloric diet
Xu et al., 2022 [46]	48 M&F	Intermittent-CR + Exe Continuous-CR + Exe Exe alone	4	VO_2max_ BFP BMI	Overweight	Intermittent-CR + Exe: 21.3 ± 2.24 Continuous-CR + Exe: 21.3 ± 2.24 Exe alone: 21.3 ± 2.24	Intermittent-CR + Exe: 26.90 ± 1.46 Continuous-CR + Exe: 26.39 ± 1.63 Exe alone: 26.12 ± 2.01	Participants in the Intermittent-CR group consumed 30% of their daily recommended energy intake (approximately 500–1000 kcal) on two non-consecutive days per week, while eating ad libitum on the remaining five days. The Continuous-CR group followed a daily hypoenergetic diet, consuming 70% of their estimated energy requirements—approximately 1300–1600 kcal for females and 1600–1900 kcal for males.	Participants in the Exe alone group consumed their full daily recommended energy intake, distributed across three meals.	Participants completed exercise sessions five times per week, consisting of high-intensity intervals at 80% of their VO_2max_ interspersed with active recovery periods at 50% VO_2max_. Each session included five 3 min cycling bouts, totaling 30 min of exercise, preceded by a 10 min warm-up and followed by a 10-min cool-down. The HIIT protocol was structured to create an approximate energy deficit of 310 kcal per session, with session duration individually adjusted.	The daily recommended energy intakes were 1800–2250 kcal for females and 2250–2700 kcal for males	Hypocaloric diet
Yassine et al., 2009 [37]	24 M&F	CR + Exe Exe alone	12	VO_2max_ FM FFM BMI BW	Older adults with obesity	CR + Exe: 65.5 ± 5 Exe alone: 65.5 ± 5	CR + Exe: 33.7 ± 4.7 Exe alone: 35.3 ± 5.8	In the CR + Exe group, participants were instructed to decrease their daily energy intake by 500 kcal.	NR	Participants engaged in exercise sessions lasting 50 to 60 min per day, five days per week. The regimen included treadmill walking and/or cycling on an ergometer, with over 75% of the effort dedicated to treadmill activity. During the initial sessions, exercise intensity was set at 60% to 65% of HRmax, increasing to 80% to 85% HRmax (~70% VO_2max_) after week 4.	Participants in the Exe + CR group consumed fewer calories (1303 ± 210 kcal/day) than those in the Exe group (2141 ± 1338 kcal/day)	Hypocaloric diet

Abbreviations: F: Female, M: Male, Exe: Exercise, CR: Calorie restricted, CON: Control, kcal: Kilocalorie, d: day, Min: Minute, g: gram, MJ: Megajoules, h: hour, NR: No report, ND: No diet, IF: Intermittent fasting, TRF: Time restricted feeding, ADF: Alternate day fasting, RIF: Ramadan intermittent fasting, RM: Rep max, HR: Heart rate, VO_2max_: Maximal oxygen consumption, SJ: squat jump, CMJ: Counter movement jump, HIIT: High intensity interval training, SIT: Sprint interval training, GSSG: Small sided games group, GRSA: Repeated sprint ability group, HRR: Heart rate reserve, BMI: Body mass index, FM: Fat mass, FFM: Fat-free mass, BFP: Body fat percentage.

## Data Availability

All data used in the analyses are available on request to the corresponding author.

## Supplementary Materials

The following supporting information can be downloaded at: https://www.mdpi.com/article/10.3390/nu17121992/s1, Table S1: Search strategy; Table S2: Quality of studies assessment.
