# Supplementary material for: Effects of Intermittent Fasting and Calorie Restriction on Exercise Performance: A Systematic Review and Meta-Analysis
Source: Nutrients. 2025 Jun 13;17(12):1992. doi: 10.3390/nu17121992 (PMC12195750; doi:10.3390/nu17121992)
Supplement: Supplementary file 1 [file nutrients-17-01992-s001.zip › nutrients-3683287-supplementary.pdf]

Supplementary Table S1. Search strategy

Databases	Search strategy	Limits	Results
PubMed	(("Fasting"[Title/Abstract] OR "Ramadan"[Title/Abstract] OR "Intermittent Fasting"[Title/Abstract] OR "Intermittent Energy Restriction"[Title/Abstract] OR "Intermittent caloric Restriction"[Title/Abstract] OR "Caloric Restriction"[Title/Abstract] OR "Calorie Restriction"[Title/Abstract] OR "Time-Restricted Feeding"[Title/Abstract] OR "Time Restricted Feeding"[Title/Abstract] OR "TRF"[Title/Abstract] OR "Time-Restricted Eating"[Title/Abstract] OR "Time Restricted Eating"[Title/Abstract] OR "TRE"[Title/Abstract] OR "Alternate-Day Fasting"[Title/Abstract] OR "Alternate Day Fasting"[Title/Abstract] OR "ADF"[Title/Abstract] OR "Periodic Fasting"[Title/Abstract] OR "fasted state"[Title/Abstract]) AND ("performance"[Title/Abstract] OR "repeated sprint"[Title/Abstract] OR "VO_2max"[Title/Abstract] OR "oxygen consumption"[Title/Abstract] OR "VO_2peak"[Title/Abstract] OR "speed"[Title/Abstract] OR "muscle strength"[Title/Abstract] OR "exercise performance"[Title/Abstract] OR "athletic performance"[Title/Abstract] OR "sports performance"[Title/Abstract] OR "strength"[Title/Abstract] OR "force"[Title/Abstract] OR "muscular strength"[Title/Abstract] OR "muscular force"[Title/Abstract] OR "power"[Title/Abstract] OR "anaerobic"[Title/Abstract] OR "anaerobic power"[Title/Abstract] OR "anaerobic performance"[Title/Abstract] OR "anaerobic capacity"[Title/Abstract] OR "aerobic"[Title/Abstract] OR "aerobic capacity"[Title/Abstract] OR "aerobic power"[Title/Abstract] OR "aerobic performance"[Title/Abstract] OR "endurance"[Title/Abstract] OR "endurance capacity"[Title/Abstract] OR "endurance power"[Title/Abstract] OR "endurance performance"[Title/Abstract] OR "fatigue"[Title/Abstract] OR "recovery"[Title/Abstract] OR "exercise"[Title/Abstract] OR "training"[Title/Abstract] OR "exercise training"[Title/Abstract] OR "physical activity"[Title/Abstract])) AND ("Controlled trial"[Title/Abstract] OR "randomized"[Title/Abstract] OR "random"[Title/Abstract] OR "randomly"[Title/Abstract] OR "randomized clinical trial"[Title/Abstract] OR " Randomized controlled trial"[Title/Abstract] OR "RCT"[Title/Abstract] OR "blinded"[Title/Abstract] OR "double blind"[Title/Abstract] OR "double blinded"[Title/Abstract] OR "trial"[Title/Abstract] OR "controlled clinical trial"[Title/Abstract] OR "clinical trial"[Title/Abstract] OR "clinical trials"[Title/Abstract] OR "crossover procedure"[Title/Abstract] OR "cross-over trial"[Title/Abstract] OR "double blind procedure"[Title/Abstract] OR "equivalence trial"[Title/Abstract])	Humans, English	4242
Scopus	(("Fasting" or "Ramadan" or "Intermittent Fasting" or "Intermittent Energy Restriction" or "Intermittent caloric Restriction" or "Caloric Restriction" or "Calorie Restriction" or "Time-Restricted Feeding" or "Time Restricted Feeding" or "TRF" or "Time-Restricted Eating" or "Time Restricted Eating" or "TRE" or "Alternate-Day Fasting" or "Alternate Day Fasting" or "ADF" or "Periodic Fasting" or "fasted state") AND ("performance" or "repeated sprint" or "VO_{2max} " or "oxygen consumption" or "VO_2peak" or "speed" or "muscle strength" or "exercise performance" or "athletic performance" or "sports performance" or "strength" or "force" or "muscular strength" or "muscular force" or "power" or "anaerobic" or "anaerobic power" or "anaerobic performance" or "anaerobic capacity" or "aerobic" or "aerobic capacity" or	Article, English	11083

	"aerobic power" or "aerobic performance" or "endurance" or "endurance capacity" or "endurance power" or "endurance performance" or "fatigue" or "recovery" or "exercise" or "training" or "exercise training" or "physical activity")) AND ("Controlled trial" or "randomized" or "random" or "randomly" or "randomized clinical trial" or " Randomized controlled trial" or "RCT" or "blinded" or "double blind" or "double blinded" or "trial" or "controlled clinical trial" or "clinical trial" or "clinical trials" or "crossover procedure" or "cross-over trial" or "double blind procedure" or "equivalence trial")		
Web of science	((TS=("Fasting" or "Ramadan" or "Intermittent Fasting" or "Intermittent Energy Restriction" or "Intermittent caloric Restriction" or "Caloric Restriction" or "Calorie Restriction" or "Time-Restricted Feeding" or "Time Restricted Feeding" or "TRF" or "Time-Restricted Eating" or "Time Restricted Eating" or "TRE" or "Alternate-Day Fasting" or "Alternate Day Fasting" or "ADF" or "Periodic Fasting" or "fasted state")) AND TS=("performance" or "repeated sprint" or " VO_{2max}" or "oxygen consumption" or "VO_2peak" or "speed" or "muscle strength" or "exercise performance" or "athletic performance" or "sports performance" or "strength" or "force" or "muscular strength" or "muscular force" or "power" or "anaerobic" or "anaerobic power" or "anaerobic performance" or "anaerobic capacity" or "aerobic" or "aerobic capacity" or "aerobic power" or "aerobic performance" or "endurance" or "endurance capacity" or "endurance power" or "endurance performance" or "fatigue" or "recovery" or "exercise" or "training" or "exercise training" or "physical activity")) AND TS=("Controlled trial" or "randomized" or "random" or "randomly" or "randomized clinical trial" or " Randomized controlled trial" or "RCT" or "blinded" or "double blind" or "double blinded" or "trial" or "controlled clinical trial" or "clinical trial" or "clinical trials" or "crossover procedure" or "cross-over trial" or "double blind procedure" or "equivalence trial")	Article, English	6583

Supplementary Table S2. Quality of studies assessment

Authors and Year of Publication	1	2	3	4	5	6	7	8	9	Total score
Ballor et al. 1988 [57]	×	✓	×	×	×	✓	×	✓	✓	4
Batitucci et al. 2022 [58]	✓	✓	×	✓	×	×	×	✓	✓	5
Bouhlel et al, 2013 [35]	×	✓	×	✓	×	✓	×	✓	✓	5
Brady et al. 2021 [47]	✓	✓	×	✓	×	×	×	✓	✓	5
Brini et al. 2018 [48]	✓	✓	×	✓	×	✓	×	✓	✓	6
Brini et al. 2020 [49]	✓	✓	×	✓	×	✓	×	✓	✓	6
Brini et al. 2021 [50]	✓	✓	×	✓	×	✓	×	✓	✓	6
Brisswalter et al, 2011 [51]	×	×	×	✓	✓	✓	×	✓	✓	5
Cho et al. 2019 [63]	✓	✓	×	✓	✓	×	×	✓	✓	6
Christiansen et al. 2010 [38]	✓	✓	×	×	×	✓	×	✓	✓	5
Coker et al. 2009 [39]	✓	✓	×	✓	×	✓	×	✓	✓	6
Cooke et al. 2022 [40]	✓	✓	×	✓	×	✓	✓	✓	✓	7
Correia et al. 2023 [52]	✓	✓	×	✓	×	✓	×	✓	✓	6
Correia et al. 2024 [31]	✓		×	✓	×	✓	×	✓	✓	6
Cox et al. 2004 [53]	✓	✓	×	×	×	×	×	✓	✓	4

Habermann et al. 2015 [59]	✓	✓	✗	✓	✗	✗	✗	✓	✓	5
Hagan et al. 1986 [41]	✓	✓	✗	✓	✗	✓	✗	✓	✓	6
Haganes et al. 2022 [60]	✓	✓	✗	✓	✗	✗	✓	✓	✓	6
Hammer et al. 1989 [61]	✓	✓	✗	✓	✗	✗	✗	✓	✓	5
Jefferson et al. 2015 [42]	✓	✓	✗	✓	✗	✓	✗	✓	✓	6
Kirkendall et al. 2008 [33]	✗	✓	✗	✓	✗	✓	✗	✓	✓	5
Kotarski et al. 2021 [43]	✓	✓	✗	✓	✗	✓	✗	✓	✓	6
Moro et al. 2016 [23]	✓	✓	✗	✓	✓	✓	✗	✓	✓	7
Moro et al. 2020 [36]	✓	✓	✗	✓	✓	✓	✗	✓	✓	7
Moro et al. 2021 [54]	✓	✓	✗	✓	✓	✓	✗	✓	✓	7
Nicklas et al. 2015 [44]	✓	✓	✗	✓	✓	✓	✗	✓	✓	7
Nicklas et al. 2018 [32]	✓	✓	✗	✓	✓	✓	✗	✓	✓	7
Nieman et al. 2000 [62]	✓	✓	✗	✓	✗	✓	✗	✓	✓	6
Stratton et al. 2020 [55]	✓	✓	✗	✓	✗	✗	✗	✓	✓	5
Tinseley et al. 2016 [22]	✓	✓	✗	✓	✗	✗	✗	✓	✓	5
Tovar et al. 2019 [56]	✓	✓	✗	✓	✗	✗	✗	✓	✓	5
Wang et al. 2018 [34]	✓	✓	✗	✓	✗	✓	✗	✓	✓	6
Weiss et al. 2016 [45]	✓	✓	✗	✓	✗	✓	✗	✓	✓	6
Xu et al. 2022 [46]	✓	✓	✗	✓	✓	✓	✗	✓	✓	7
Yassine et al. 2009 [37]	✓	✓	✗	✗	✗	✓	✗	✓	✓	5
